# Rapid synergistic thrombolysis of ischemic stroke guided by high-resolution and high-speed photoacoustic cerebrovascular imaging

**DOI:** 10.1016/j.pacs.2025.100722

**Published:** 2025-04-04

**Authors:** Mengtao Han, Zhiwei Xue, Mengchen Yu, Nanlin You, Yaguang Ren, Zhiqiang Xu, Zhifeng Wu, Yiming He, Zonghai Sheng, Chengbo Liu, Donghai Wang, Jingqin Chen

**Affiliations:** a Department of Neurosurgery, Qilu Hospital of Shandong University, Cheeloo College of Medicine and Institute of Brain and Brain-Inspired Science, Shandong University, Jinan 250012, China; b Research Center for Biomedical Optics and Molecular Imaging, Shenzhen Institutes of Advanced Technology, Chinese Academy of Sciences, Shenzhen 518055, China; c State Key Laboratory of Biomedical Imaging Science and System, Shenzhen 518055, China; d Shandong Key Laboratory of Brain Health and Function Remodeling, Jinan 250012, China; e Department of Neurosurgery, Qilu Hospital of Shandong University Dezhou Hospital, Dezhou 253000, China; f Research Center for Advanced Detection Materials and Medical Imaging Devices, Institute of Biomedical and Health Engineering, Shenzhen Institutes of Advanced Technology, Chinese Academy of Sciences, Shenzhen 518055, China

**Keywords:** Ischemic stroke, Photoacoustic imaging, Rapid thrombolysis, Cerebrovascular imaging, Biomimetic drug delivery system

## Abstract

Thrombosis is the major cause of ischemic stroke and poses a serious health burden globally. Current thrombolytic strategies, such as systematic administration of recombinant human tissue plasminogen activator (rt-PA), are challenged by limited thrombolysis efficiency due to low targeting ability and a short plasma half-life. Here, we report a rapid synergistic strategy that integrates sonothrombolysis and rt-PA mediated pharmacological thrombolysis to achieve accurate and efficient treatment of ischemic stroke. The strategy (PLPA@PFP) uses a platelet-biomimetic membrane as a carrier to deliver both perfluoropentane (PFP) and rt-PA, prolonging half-life and effectively accumulating at the thrombus within 0.5 hours. Upon exposure to focused ultrasound, PFP-based cavitation effects significantly enhance thrombus breakdown and rt-PA penetration, enabling synergistic sono/pharmacological thrombolysis both *in vitro* and *in vivo*. High-resolution photoacoustic (PA) imaging provides direct assessment of vascular reperfusion following therapeutic intervention in a murine model of ischemic stroke, offering important guidance for clinical treatment.

## Graphical Abstract

**Figure:**
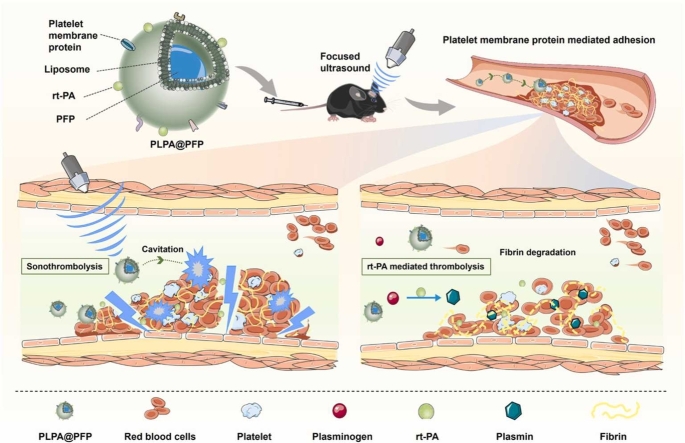

## 1 Introduction

Thrombosis is caused by a disruption in hemostatic regulation, which can block blood flow and lead to life-threatening cerebrocardiovascular diseases, such as ischemic stroke, myocardial infarction, and pulmonary embolism[1]. Among them, ischemic stroke is the second leading cause of death worldwide[2]. Despite significant advances in diagnosis and treatment modalities, the morbidity and mortality rates related to stroke remain alarmingly high[3], [4]. Once a stroke occurs, it is estimated that delaying acute stroke reperfusion by 1 h would result in the loss of approximately 120 million neurons, equivalent to the neuronal loss over 3.6 years of normal aging[5], [6], [7]. Clinical reports have demonstrated that if the diagnosis and treatment of stroke were completed within a 4.5–6 h window, the therapeutic benefit could be maximized[7], [8]. Therefore, rapid diagnosis and thrombolysis are critical following stroke.

Traditional medical imaging modalities such as computed tomography (CT) and magnetic resonance imaging (MRI) provide direct diagnosis tools for stroke and assessment of treatment eligibility[9]. CT scans are fast, typically completed within 10 s[10], but they involve ionizing radiation and have limited sensitivity in detecting ischemic lesions[11]. Magnetic resonance diffusion-weighted imaging is highly sensitive for detecting acute ischemic stroke, but its high cost and longer acquisition time can delay emergency treatment[12], [13], [14], [15]. In recent years, photoacoustic (PA) imaging has emerged as a cutting-edge medical imaging modality that uses laser pulses to induce thermoelastic expansion in tissues, generating ultrasound waves that reflect tissue-specific light absorption[16], [17]. It combines the dual advantages of optical and ultrasonic imaging, achieving micron-level imaging resolution and centimeter-level imaging depth[18], [19]. Due to the strong visible-light absorption of hemoglobin, PA imaging can acquire structural information of blood vessels without the need for contrast agents[20], [21]. Additionally, it can differentiate the absorption spectra of oxygenated and deoxygenated hemoglobin, enabling precise quantification of oxygen saturation (sO₂)[22]. Thus, PA imaging provides a promising method for the rapid and accurate diagnosis of stroke, while also guiding effective thrombolysis by assessing cerebrovascular structure and function.

For thrombolytic therapy in stroke, the recombinant human tissue plasminogen activator (rt-PA) is extensively used in clinical practice[1], [4]. However, free rt-PA lacks thrombus specificity and is limited by short half-life, potentially leading to thrombolysis failure and undesirable bleeding complications[23], [24], [25], [26]. To overcome these limitations, various nanocarriers have been developed to enhance rt-PA delivery [27], [28], [29]. Among these, biomimetic drug delivery systems (BDDSs) that use biological components to improve the efficacy and targeting of rt-PA have gained attention due to their superior biocompatibility and lower immunogenicity[30], [31], [32]. Platelets (PLTs) play a crucial role in thrombus formation. When blood vessels are injured, PLTs are the first responders, quickly adhering to exposed collagen through membrane receptors like glycoprotein Ⅵ (GPⅥ) and integrins[31]. Inspired by this, PLT membranes have been explored as a biomimetic drug carrier for rt-PA, demonstrating enhanced targeting, prolonged circulation time and reduced cerebral damage[32]. However, a significant challenge remains, as the dense structure of thrombi often impedes the penetration of rt-PA, reducing the thrombolytic efficiency. Therefore, promoting rt-PA penetration within thrombi is essential for achieving rapid and effective thrombolysis.

Sonothrombolysis, a physical method that uses continuous US and sonographic contrast agent microbubbles (MBs) to disrupt the clot, has been proven to increase the recanalization rate and improve the prognosis of patients with ischemic stroke[33], [34]. MBs lower the cavitation threshold, facilitating clot disruption through stable and inertial cavitation. Stable cavitation induces MBs oscillation and microstreaming, which loosens clots and enhances rt-PA penetration[33], [35], [36], [37]. In contrast, inertial cavitation, occurring at higher pressure, results in rapid MB expansion and collapse, generating microjets that further aid clot disruption. Thus, sonothrombolysis offers a rapid and controllable method for clot disruption. However, MBs-mediated sonothrombolysis is limited by the short circulation time and rapid clearance of MBs[38], [39]. Recently, phase-change nanodroplets (NDs) have shown superior properties, including prolonged circulation time, enhanced tissue penetration, and the production of smaller clot debris, which reduces the risk of embolism[40], [41], [42], [43], [44]. Consequently, NDs-based sonothrombolysis holds great potential for promoting the penetration of rt-PA within thrombi.

**Fig. 1:**
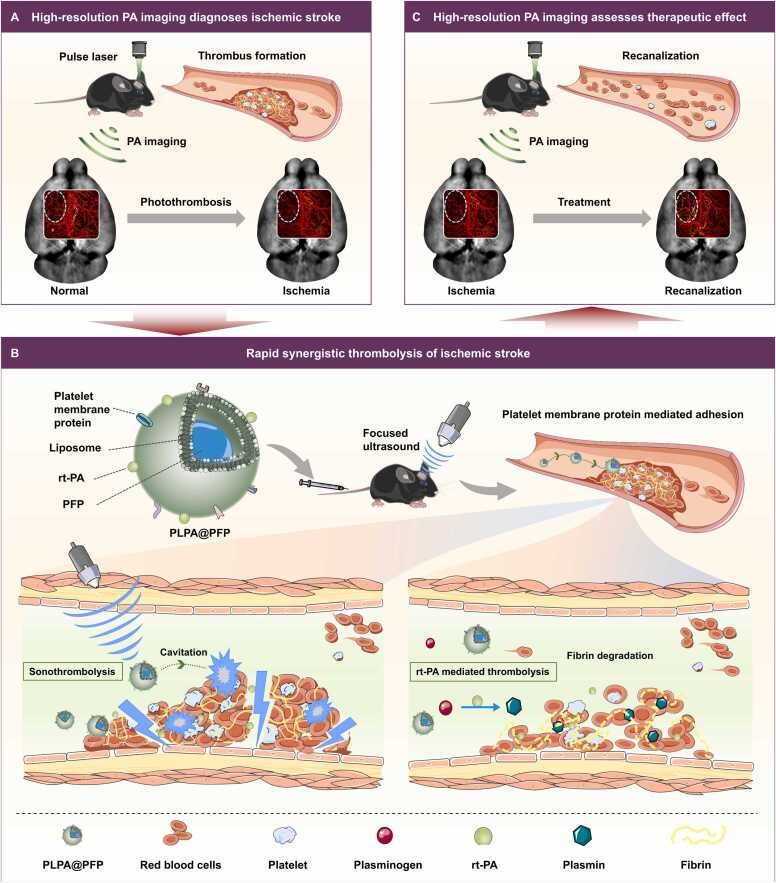
Schematic illustration of photoacoustic imaging-guided physical/pharmacological rapid synergistic thrombolysis.

In this study, we developed a synergistic thrombolysis strategy by applying NDs-assisted sonothrombolysis and rt-PA-mediated pharmacological thrombolysis (Fig. 1). In this strategy, the PLT membrane hybridized with liposome was prepared as a BDDS of perfluoropentane (PFP) and rt-PA (PLPA@PFP), which showed good biocompatibility and a thrombus-targeted effect *in vitro* and *in vivo*. The small size of PLPA@PFP facilitated its delivery to thrombus compared to traditional MBs. Combined with focused US, PLPA@PFP demonstrated a synergistic and rapid thrombolytic effect *in vitro* and *in vivo*. High-resolution and high-speed PA imaging and laser speckle imaging enabled complementary, non-invasive monitoring and assessment of cerebrovascular structures, blood oxygenation, and blood flow during thrombolysis treatment *in vivo*. Together, this work not only provides new rapid thrombolysis strategy, but also opens up new avenues for therapeutic effect evaluation of ischemic stroke.

## 2 Experimental section

### 2.1 Preparation of liposomes

Liposomes were prepared by thin film-hydration and extrusion method. Briefly, 18 mg of 2-dipalmitoyl-sn-glycero-3-phosphocholine (DPPC), 3.5 mg of 1,2-distearoyl-sn-glycero-3-phosphoethanolamine-N-[poly(ethylene glycol)]-2000 (DSPE-PEG2000), 1 mg of 1,2-dipalmitoyl-sn-glycero-3-phosphate (DPPA), 0.5 mg of 1,2-distearoyl-sn-glycero-3-phosphoethanolamine-N-[carboxy(polyethylene glycol)-2000] (DSPE-PEG2000-COOH) was dissolved in 5.0 mL of chloroform, and then evaporated at 65 °C with a rotary evaporating system to get a thin lipid film. The lipid film was hydrated with 5 mL ddH_2_O and the suspension was sonicated in an ice bath for 20 min and then extruded through 0.4 and 0.2μm polycarbonate membranes (Whatman, Maidstone, UK) at room temperature using an extruder system (Avanti Polar Lipids, Alabaster, AL, USA). The liposomes were stored at 4 °C until use.

### 2.2 Extraction of platelet membrane

Platelet membrane was prepared according to previous studies described[31]. Firstly, whole blood of C57BL/6 J mice was obtained from the orbital veins using 0.2 μm capillary tubes and anticoagulated with 5 mM ethylenediaminetetraacetic acid (EDTA). Then, the blood was centrifuged at 100 g for 20 min to get upper platelet-rich plasma, which would be collected and transferred to another tube. To prevent platelet activation, 2 µM prostaglandin E1 would be added and then centrifuged at 850 g for 20 min to obtain pure platelets. After carefully removing the supernatant, platelets were resuspended in PBS containing 1 mM EDTA and a protease inhibitor. To extract the platelet membrane, suspensions were repeatedly snap-frozen with liquid nitrogen and thawed to room temperature at least three times. They were then sonicated for 5 minutes using a 100 W, 42 kHz sonicator. The membranes were pelleted by centrifugation at 4000 g for 15 minutes at 4°C and washed three times with PBS containing protease inhibitors. Finally, the purified platelet membranes were stored at −80°C.

### 2.3 Membrane fusion and rt-PA conjugation

500 μL of liposomes and platelet membranes at a mass ratio of 10:1 were placed into a capped vial and sonicated using a 130 W, 20 kHz sonicator for 30 s. Subsequently, the size of the hybrid platelet-liposomes (PL) was adjusted to less than 200 nm using extrusion equipment and membrane filters. For rt-PA conjugation to PL, 2 mM EDC (Pierce™ EDC No-Weigh Format, CAS # 25952–53–8, Thermo Scientific, USA) and 5 mM Sulfo-NHS (No-Weigh™ Format, CAS # 106627–54–7, Thermo Scientific, USA) were added to 1 mL of PL. The reaction mixture was incubated for 15 minutes at room temperature to activate carboxylates. Then, 20 mM of 2-mercaptoethanol was added to quench the excess EDC, and rt-PA was introduced at an equal molar ratio with DSPE-PEG2000-COOH in PL, reacting for 2 hours at room temperature. Subsequently, 10 mM hydroxylamine was added to quench the reaction, and the excess quenching reagent was removed by dialysis. Finally, the suspension was mixed with 50 μL PFP and sonicated with a 100 W, 20 kHz sonicator for 45 seconds to obtain PLPA@PFP.

### 2.4 Characterization of PLPA@PFP

The morphology of PLPA and PLPA@PFP was analyzed using TEM after negative staining with a 2 % (w/w) sodium phosphotungstate solution. UV-Vis-NIR absorption spectra were measured using a Shimadzu UV-3600 spectrophotometer (Japan). The particle size, polydispersity index, and zeta potential were determined through dynamic light scattering (DLS) using a Zetasizer Nano ZS90 (Malvern, U.K.). Elemental mapping of PLPA@PFP was conducted via energy-dispersive X-ray (EDX) analysis (Thermo Fisher, USA). To confirm membrane fusion, platelet membranes were labeled with 10 μM Dil dye (Beyotime, China) and liposomes with 5 μM Dio dye for 15 minutes at room temperature during preparation. Confocal microscopy (Leica TCS SP8 CARS, Germany) was utilized to detect the fluorescence signals of the nanoparticles. Protein content in all samples was quantified using Coomassie staining as described in a previous report[45].

### 2.5 In vitro thrombus targeting of PLPA@PFP

To trace the nanoparticles of this study, rt-PA, LPA@PFP and PLPA@PFP were labeled with ICG or Dio. *In vitro* binding assays were performed to determine whether PLPA@PFP could actively target thrombus, inflamed endothelial cells, fibrinogen and collagen Ⅳ, and activated platelets. In order to determine the binding ability of PLPA@PFP to thrombus, the artificial blood clots were prepared. Fresh blood was collected from the orbital vein of male C57/BL6J mice and 100 µL of blood was divided into 1.5 mL polypropylene centrifuge tubes, each containing 50 U of thrombin solution. The tubes were incubated at 37°C for 2 hours and then moved to 4°C overnight to form solid clots. The blood clots were subsequently transferred to vials containing 1 mL of saline. Following this, 100 μL of saline, rt-PA, LPA@PFP, or PLPA@PFP were added to the respective tubes and incubated for 15 minutes. The blood clots were then removed and washed three times with PBS. The fluorescence of the thrombus clots was analyzed using the in vivo imaging *system (*IVIS) spectrum (PerkinElmer, USA).

To assess the targeting ability of PLPA@PFP to inflamed endothelial cells, HUVECs were seeded in confocal dishes at a density of 1 × 10⁵ cells per well. After the cells reached 80 % confluence, they were treated with LPS at a concentration of 100 ng/mL and incubated for 24 hours to induce vascular inflammation. The cells were then fixed with 4 % paraformaldehyde at room temperature for 30 minutes and washed several times with PBS. ICG-labeled PLPA@PFP or LPA@PFP were added to the dishes, incubated for 30 minutes at 37°C, and washed several times with PBS. The fluorescence of different groups was detected by a laser-scanning confocal microscope.

To investigate the fibrin/ collagen Ⅳ-binding property of PLPA@PFP, 100 μL fibrinogen (10 mg/mL) was coated to slides and incubated at 37℃ for one night. Then, the slides were incubated with Dio-labeled PLPA@PFP or LPA@PFP for 30 min at 37 °C and washed with PBS at least 3 times. The fluorescence of Dio was detected and quantified. The *in vitro* targeting ability of PLPA to activated platelets was examined using flow cytometry. Platelets were pre-activated with 2 U/mL thrombin and 8 nM CaCl₂. The activated or inactivated platelets were then incubated with ICG-labeled LPA and PLPA for 30 minutes at 37°C and washed three times with PBS. The fluorescence signals were then detected by a flow cytometer (Beckman CytoFLEX S, USA).

### 2.6 Cell culture

HUVECs were obtained from the Cell Bank of the Chinese Academy of Sciences. They were cultured in Dulbecco's modified eagle medium (DMEM) containing 10 % fetal bovine serum (FBS) and 1 % penicillin-streptomycin, and placed in an incubator at 37 ℃ with 5 % CO_2_. The culture media were replaced every 2 days.

### 2.7 Photochemically-induced stroke models

All animal experiments were performed according to the Guidelines of Animal Care and Use Committee of Shenzhen Institute of Advanced Technology, Chinese Academy of Sciences (SIATACUC), and were conducted in compliance with protocols approved by the SIATACUC (SIAT-IACUC-240105-YGS-CJQ-A2423). The photochemically-induced acute ischemic stroke model was established according to the previous protocol[46], [47]. Briefly, male mice (8–10weeks, 25–30 g, Beijing Vital River Laboratory Animal Technology Co., Ltd) were anesthetized with 1 % isoflurane. After making a midline incision in the skin and removing the connective tissue, the intact skull was exposed. Then, Rose Bengal was injected via the tail vein at a dose of 100 mg/kg. Subsequently, a 532 nm laser beam with a 1.5 mm diameter was positioned at 1 mm posterior to the left coronal suture for 5 minutes and illuminated for 5 min. Finally, the scalp was sutured, and the stroke model was induced. All the mice were irradiated with a uniform 250 mW laser and positioned at the same height.

### 2.8 In vivo thrombus targeting ability of PLPA@PFP

To assess the *in vivo* targeting effect of the nanoparticles, acute ischemic stroke model mice were anaesthetized and injected intravenously with saline and ICG-labeled rt-PA, PLPA@PFP and LPA@PFP respectively. Throughout 24 h, the fluorescence signal of different time points at the infarct site of the brain was determined by an IVIS spectrum.

### 2.9 Colocalization of thrombus and nanoparticles

After 2 h of being illuminated, model mice were injected intravenously with saline and ICG-labeled rt-PA, PLPA@PFP and LPA@PFP. Mice were sacrificed 2 h after administration, and perfusion was carried out using cold phosphate-buffered saline (PBS), followed by 4 % paraformaldehyde (PFA). The brain samples were then removed and fixed with PFA overnight at 4°C. After dehydrated with 30 % sucrose solution and embedded with optimal cutting temperature compound (OCT), the samples were sliced into 25μm sections by a freezing microtome (Leica CM 1950, Germany). Subsequently, the prepared sample slices were placed at room temperature and washed with PBS for immunofluorescence staining. The slices were firstly blocked with 30 % goat serum for 1 h. Then, they were incubated with primary antibodies of rat anti-CD31 (1:1000, Abcam, ab256569) and rabbit fibrinogen antibody (1:1000, Abcam, ab92572) overnight at 4°C. Secondary antibodies of goat anti-rat IgG dylight 488 (1:1000, Abbkine) and goat anti-rabbit IgG dylight 594 (1:1000, Abbkine) were used at room temperature for 1 h. Then, all the samples were washed with PBS. Finally, nuclei were stained with DAPI (Sigma). All samples were examined using a confocal microscope (Leica TCS SP8 CARS, Germany). Fibrinogen antibody was used to detect fibrin deposition in thrombus formation, which has been validated in previous studies[48], [49], [50].

### 2.10 Determination of fibrinolytic activity of PLPA@PFP

The fibrinolytic activity of PLPA@PFP was determined by thrombolysis of blood clots, which was prepared as described above. The prepared blood clots were dried and weighted firstly. Then, they were placed into centrifuge tubes containing 1 mL saline. Then, 100ul of saline, PL@PFP, rt-PA, and PLPA@PFP with the equivalent concentration of rt-PA (10ug/mL) were added into the solution and incubated at 37°C. For the PL@PFP and PLPA@PFP treatment groups, a focused ultrasound probe was positioned on the tubes, and ultrasound was applied for 10 minutes. The ultrasound treatment was performed using a focused ultrasound transducer (Valpey Fisher Inc., Hopkinton, MA, USA) with a diameter of 50 mm and a focal length of 35 mm, operating at a frequency of 0.5 MHz. The lateral focal size was approximately 2.2 mm, and the axial focal size was around 5.8 mm. The transducer was powered by an ultrasound neurostimulation instrument (Shenzhen Institute of Advanced Technology, Chinese Academy of Sciences) configured with the following settings: acoustic pressure of 0.3 MPa, 10 ms bursts, 1 Hz repetition rate and duty cycle of 50 %. The thrombolytic effect was determined by measuring OD540 of the supernatants of all sample at different time points. After 12 h, the weight of the blood clots was recorded again, and the clot lysis efficiencies were calculated.

### 2.11 Assessment of thrombolysis efficacy in a mouse model

Femoral vein thrombosis was induced as previously reported. Briefly, C57/BL6 mouse was anesthetized with isoflurane inhalation. The right femoral vein was exposed, and a filter paper soaked with 15 % ferric chloride solution was placed on the surface of the femoral vein vessel for 3 min. After removing the filter paper, the induced femoral vein thrombus was observed, and the vessel was washed with sterile PBS. To assess thrombolytic efficiency *in vivo*, 100 μL of saline, PL@PFP, rt-PA, and PLPA@PFP with an equivalent amount of rt-PA was intravenously injected. For the PL@PFP and PLPA@PFP treated groups, a focal ultrasound probe was placed above the thrombosis, and coupling gel was applied. Focused ultrasound (parameters same as above) was applied for 10 minutes. After treatment, the incision was sutured and reopened 12 h later to observe vascular recanalization. Finally, the mice were sacrificed, and femoral veins were excised and collected for HE staining to measure clot areas. Thrombolytic efficiency was determined by the area ratio of vascular occlusion to total vasculature using ImageJ software.

### 2.12 Assessment of focal cerebral blood flow (CBF), infarction volume and BBB permeability in stroke model

Acute ischemic stroke model was established in adult mice as previously described. Mice of the sham group underwent the same procedures, except for laser illumination. Two hours after inducing the stroke, saline, rt-PA, PL@PFP, or PLPA@PFP were administered via the caudal vein. Focal CBF was monitored at different time intervals (pre-occlusion, 2 h post-occlusion, and 24, 72, and 120 h post-treatment) using a laser speckle imaging system (RFLSI Ⅲ, RWD Life Science Co, China). The reduction in blood flow on the ipsilateral side of the brain was calculated as the ratio of the average perfusion values of the ipsilateral and contralateral areas over 3 min. To evaluate the infarction volume, mice were sacrificed at 120 h post-treatment, and their brains were sectioned into 2 mm-thick slices. These sections were placed in a 2 % 2,3,5-triphenyltetrazolium chloride (TTC) solution for 20 min (10 min on each side) and then fixed with 4 % paraformaldehyde (PFA) solution for 30 min at room temperature. To assess BBB permeability after treatment, Evans Blue (2 % w/v) was used. All sections were photographed with a digital camera, and the infarct sizes and BBB permeability were measured using ImageJ software.

### 2.13 In vivo photoacoustic imaging

In this study, the experimental data was acquired by a custom-built optical-resolution PAM (OR-PAM) system. Our previous publications described the detailed composition of the OR-PAM system[51]. The major components of OR-PAM system included the following: (a) a 2 kHz 532-nm nanosecond-pulsed laser (GKNQL-532; Beijing Guoke Laser Co., Beijing, China) for photoacoustic signal excitation, (b) a single-element ultrasound transducer (V214-BC-RM; Olympus-NDT, Massachusetts, USA) with 50-MHz center frequency to detected the resultant photoacoustic waves, (c) a 2-channel data acquisition (DAQ) card (CS1422; Gage Applied Technologies Inc., Lockport, New York) to digitize the photoacoustic signals at a sampling rate of 200 MS/s and (d) a precision 3-axis motorized linear stage (VT-80; Physik Instrumente, Karlsruhe, Germany) to acquire the volumetric data from mechanical raster scanning. For *in vivo* experiments, the laser beam passed through a photoacoustic combiner and illuminated the sample surface, generating photoacoustic signals, which were detected by an ultrasonic transducer. The detected photoacoustic signals were amplified and digitally processed by a 200 MS/s data acquisition card. The fluency of each laser pulse on the tissue was maintained well below 20 mJ/cm² to prevent energy damage.

Blood oxygen saturation (sO₂) was calculated based on the differential optical absorption properties of oxygenated hemoglobin (HbO₂) and deoxygenated hemoglobin (Hb) at 558 nm and 532 nm. The absorption coefficients at each wavelength were decomposed into contributions from HbO₂ and Hb using their known absorption spectra. The concentrations of HbO₂ and Hb were then calculated, and sO₂ was determined using the following formula:

$${\mathrm{sO}}_{2}=\frac{C_{{\mathrm{HbO}}_{2}}}{C_{{\mathrm{HbO}}_{2}}+C_{\mathrm{Hb}}}\times 100\%$$

### 2.14 In vivo biodistribution and safety evaluation of PLPA@PFP

To study the *in vivo* distribution, ICG-labeled rt-PA and PLPA@PFP (at a dose of 1 mg/mL ICG) were injected via the tail vein into C57/BL6J mice. Blood samples (100 μL) were collected from the orbital vein and mixed thoroughly with ethylene diamine tetraacetic acid (EDTA) buffer. The fluorescence intensity of ICG in the circulation was measured at 15 min, 30 min, 1 h, 3 h, 6 h, 12 h, 24 h, and 48 h post-injection using an IVIS instrument. Major organs (brains, hearts, livers, spleens, lungs, kidneys, intestines, and pancreas) were collected at 24 h and 48 h post-injection of rt-PA and PLPA@PFP to assess biodistribution. Healthy C57/BL6J mice were randomly assigned into four groups (n = 3 per group): healthy control, saline, rt-PA, and PLPA@PFP groups. After inducing stroke using the photochemical method, 150 μL of saline, rt-PA, or PLPA@PFP were intravenously injected into the mice 2 h post-stroke. The mice were sacrificed 7 days post-injection to collect major organs (heart, liver, spleen, lung, and kidney) for hematoxylin and eosin (H&E) staining and blood for hematological examination. Hematological examinations included hepatic function (alanine aminotransferase (ALT), aspartate aminotransferase (AST)), renal function (creatinine (CRE) and urea (UREA)), and coagulation parameters (activated partial thromboplastin time (aPTT), prothrombin time (PT), thrombin time (TT), and fibrinogen (FIB)).

### 2.15 Statistical analysis

All data are expressed as mean ± standard deviation (SD). The normality of data distribution was assessed using the Shapiro-Wilk test. For comparisons between the two groups, an unpaired two-tailed Student's *t*-test was employed. For comparisons among more than two groups, one-way analysis of variance (ANOVA) followed by Tukey's multiple comparisons test was used. Statistical analyses were performed using GraphPad Prism 9.0 software. A p-value less than 0.05 was considered statistically significant.

## 3 Results and discussion

### 3.1 Preparation and characterization of PLPA@PFP

**Fig. 2:**
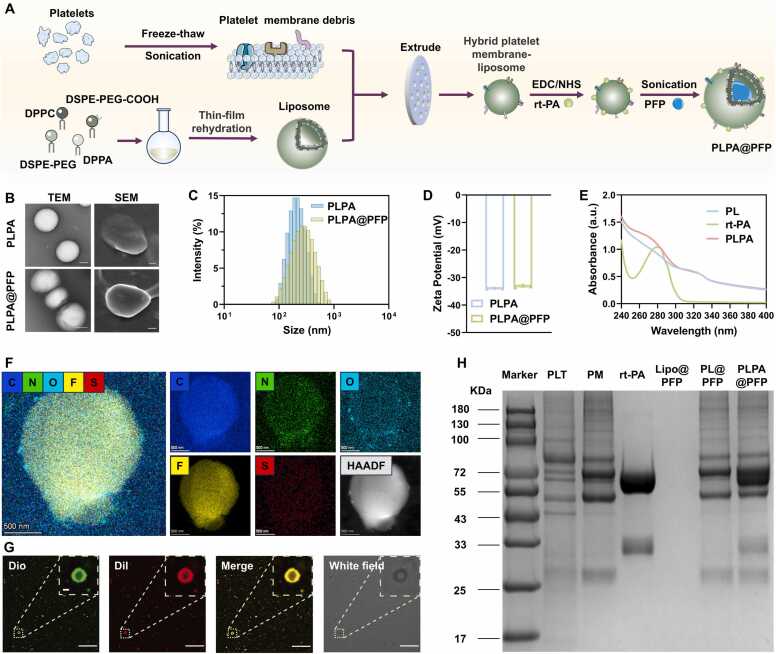
Characterization of PLPA@PFP. (A) Schematic illustration of the synthesis of PLPA@PFP. (B) TEM and SEM images of PLPA and PLPA@PFP. (C) Hydrodynamic diameters of PLPA and PLPA@PFP detected by DLS measurement. (D) Zeta potential of PLPA and PLPA@PFP. (E) UV–vis absorption spectra of rt-PA, PL, and PLPA. (F) Corresponding element mappings of C, N, O, S, and F signals and STEM of PLPA@PFP. (G) Images of PLPA@PFP under a confocal laser scanning microscope. Phospholipid labeled with Dio (green), and platelet membrane labeled with Dil (red). Scale bar = 10 µm (inside: scale bar = 500 nm). (H) SDS-PAGE analysis of proteins extracted from platelets (PLT), platelet membrane (PM), rt-PA, Lipo@PFP, PL@PFP, and PLPA@PFP.

PLPA@PFP nanoparticles were synthesized by fusing a platelet membrane with liposomes to form a hybrid cell membrane (PL), followed by chemical modification with rt-PA via covalent bond (PLPA). PFP was then loaded into PLPA via sonication, resulting in PLPA@PFP (Fig. 2A). As depicted in transmission electron microscopy (TEM) and scanning electron microscopy (SEM) images, both PLPA and PLPA@PFP exhibited uniform spherical structures (Fig. 2B). The average diameter of PLPA@PFP was 267.5 ± 11.99 nm, compared with PL (166.0 ± 1.277 nm) and PLPA (232.0 ± 16.15 nm) (Fig. 2C and Figure S1A). The zeta potential of PLPA@PFP was −33.3 ± 0.400 mV, in contrast to PL (−31.33 ± 1.850 mV) and PLPA (−34.1 ± 0.199 mV) (Fig. 2D and Figure S1B). These slight changes might be caused by rt-PA conjugation and PFP loading. Absorbance spectra of PLPA@PFP showed the characteristic peaks of both rt-PA and PL, suggesting the successful conjugation of rt-PA in PLPA (Fig. 2E). TEM elemental mappings showed the distribution of C, N, O, F, and S elements in PLPA@PFP (Fig. 2F and Figure S2). The presence of the F element demonstrated the successful loading of PFP in PLPA@PFP. In addition, we labeled liposomes with Dio and platelet membranes with Dil. The fluorescence images of PLPA@PFP displayed both Dio (green) and Dil (red) signals, verifying the successful hybridization of platelet membrane and liposomes in PLPA@PFP (Fig. 2G). The hybridization of PLT membrane and liposome could potentially reduce the use of animal blood while maintaining the targeting efficacy of thrombus and preserving desirable properties of liposome, such as stability, uniformity and suitability for mass production[52]. Coomassie’s brilliant blue assay also confirmed the coexistence of platelet membrane protein and rt-PA in PLPA@PFP, as indicated by the protein profiles in Fig. 2H. The calculated rt-PA loading efficiency of PLPA was approximately 46.48 % (Table S1), which was similar to previously reported rt-PA loading nanoparticles[32], [35]. As shown in Figure S3, no obvious changes in the diameter of PLPA in both 1X phosphate-buffered saline (PBS) and 0.5X fetal bovine serum (FBS) were seen over 120 h. After loading PFP, PLPA@PFP was stable over 3 h in 1X PBS or 0.5X FBS at room temperature in contrast-enhanced US imaging, demonstrating its good physiological stability (Figure S4).

### 3.2 *In Vitro* targeted effect of PLPA@PFP

**Fig. 3:**
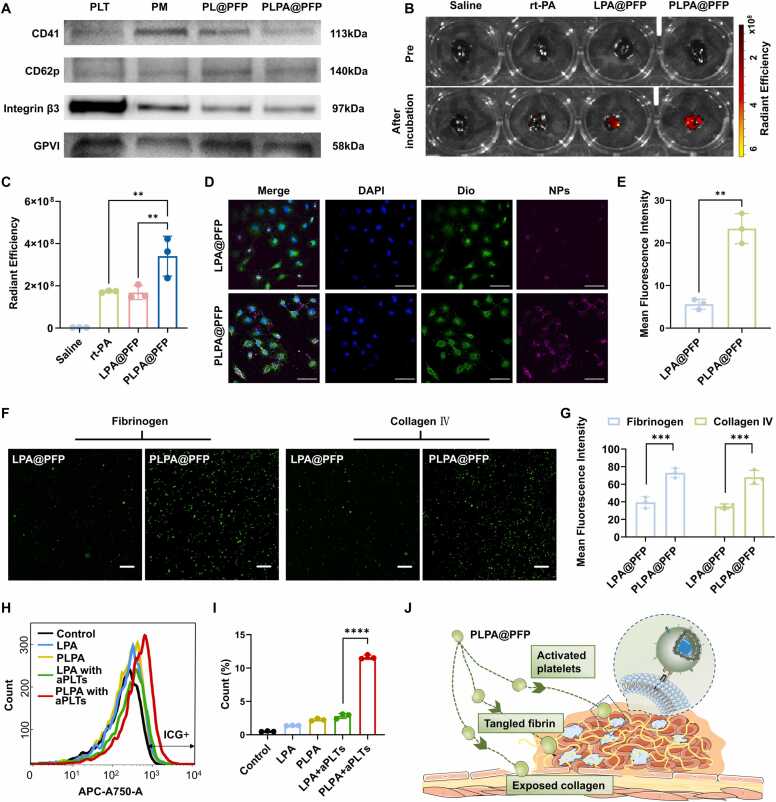
In vitro thrombus targeting ability of PLPA@PFP. (A) Representative proteins of CD41, CD62p, integrin *β*3, and GPⅥ determined using western blot. (B) Fluorescence images of artificial blood clots after incubation with saline, rt-PA, LPA@PFP, or PLPA@PFP. Nanoparticles were labeled with ICG (red signal). (C) Quantitative analysis of the relative fluorescence intensity of artificial blood clots in (B). *n* = 3, mean ± SD; * *p < 0.01. (D) Fluorescence images of LPA@PFP or PLPA@PFP retention on slides seeded with HUVECs treated with LPS. Scale bar = 100 μm. (E) Quantification analysis of LPA@PFP or PLPA@PFP retention on slides. *n* = 3, mean ± SD; * *p < 0.01. (F) Fluorescence images of LPA@PFP or PLPA@PFP retention on fibrinogen or collagen IV coated slides. Scale bar = 100 μm. (G) Quantification analysis of LPA@PFP or PLPA@PFP retention on fibrinogen- or collagen IV-coated slides. *n* = 3, mean ± SD; * **p < 0.001. (H) Representative flow cytometry histogram profiles of inactivated or activated platelets incubated with ICG-labeled LPA or PLPA. (I) Data analysis of ICG positive platelets in flow cytometry. *n* = 3, mean ± SD; * ** *p < 0.0001. (J) Schematic diagram of mechanisms of PLPA@PFP adhesion to thrombus.

As shown in western blotting analyses, PLPA@PFP retained key membrane adhesion-associated proteins, such as CD41, CD62p, Integrin β3, and glycoprotein VI (GPVI) indicating its potential for thrombus targeting (Fig. 3A). We first evaluated whether PLPA@PFP could target artificial blood clots. The blood clots were prepared by mixing fresh mouse blood with thrombin at 37°C for 2 h; these were then placed at 4℃ overnight. Well-prepared clots were incubated with saline, indocyanine green (ICG)-labeled rt-PA, LPA@PFP, or PLPA@PFP for 15 min. Clots treated with PLPA@PFP displayed a higher fluorescence signal compared with other groups (Figs. 3B and 3C). This suggested superior targeting properties of PLPA@PFP for thrombus. As endothelial cell injury is a critical trigger for thrombus formation [53], we stimulated human umbilical vein endothelial cells (HUVECs) with lipopolysaccharide (LPS) to mimic endothelial cell injury. After incubating with ICG-labeled PLPA@PFP and LPA@PFP for 30 min, the stimulated HUVECs exhibited significantly stronger fluorescence intensity in the PLPA@PFP group than that in the LPA@PFP group, suggesting excellent adhesion of PLPA@PFP to the injured endothelium (Figs. 3D and 3E).

On fibrinogen- and collagen-coated slides, the retention of PLPA@PFP was significantly increased compared with that of LPA@PFP (Figs. 3F and 3G). During the early phase of thrombus formation, activated platelets (aPLTs) upregulate various proteins and release granules to recruit leukocytes, red blood cells, and additional platelets to the site of the clot[54]. Thus, we tested whether PLPA could respond to recruitment by aPLTs. Platelets were pre-activated with 8 × 10^−9^ M Ca^2+^ and 2 U/mL of thrombin, and these were then incubated with LPA and PLPA, respectively. We found that PLPA had 4-fold higher adherence of activated platelets compared with LPA; however, their binding affinity to inactivated platelets was not obvious (Figs. 3H and 3I). Thus, PLPA could respond to the recruitment of activated platelets without interacting with circulating inactivated platelets. By selectively targeting activated platelets within the thrombus, PLPA@PFP showed the potentials for improved efficacy of thrombolysis while minimizing systemic exposure and off-target effects. The mechanisms underlying the targeting ability of PLPA@PFP (Fig. 3J) are likely ascribed to 1) the passive recruitment of platelet membrane-cloaked nanoparticles by thrombotic sites [32]; 2) the specific binding of integrin αIIbβ3 on platelet membranes to fibrinogen in blood clots [55]; and 3) the specific adhesion of GPVI on the platelet membrane with collagen of damaged endothelium[56].

### 3.3 In vivo targeting effect of PLPA@PFP in an ischemic stroke mouse model

**Fig. 4:**
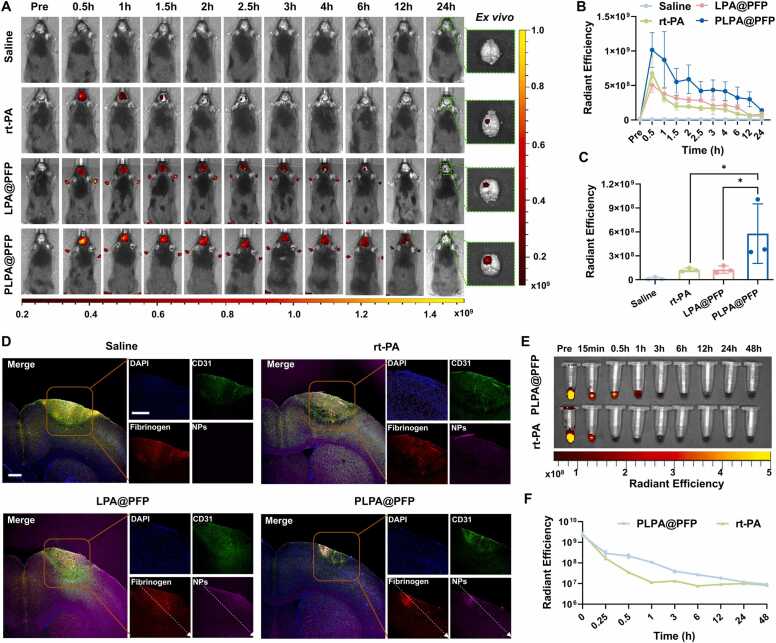
In vivo targeting of PLPA@PFP in ischemic stroke models. (A) Representative fluorescence images of the brain after injection of saline, ICG-labeled rt-PA, LPA@PFP, and PLPA@PFP within 24 h. (B) Radiant efficiency changes of saline, ICG-labeled rt-PA, LPA@PFP, and PLPA@PFP in brain infarction sites. *n* = 3, mean ± SD. (C) Semiquantitative fluorescence analysis of infarction site at 24 h. *n* = 3, mean ± SD; *p < 0.05. (D) Representative fluorescence images of thrombi in saline-, rt-PA-, LPA@PFP-, and PLPA@PFP-treated groups. Nuclei stained with DAPI (blue); blood vessels represented with CD31 (green); thrombus represented with fibrinogen/fibrin (red); rt-PA, LPA@PFP, and PLPA@PFP labeled with ICG (pink). Scale bar= 500 μm. (E) Blood fluorescence of stroke mice treated with ICG-labeled PLPA@PFP and rt-PA. (F) Data analysis of fluorescence intensity of PLPA@PFP and rt-PA in circulation. *n* = 3, mean ± SD.

Encouraged by the excellent targeted effect of PLPA@PFP *in vitro*, the efficacy of PLPA@PFP in targeting thrombotic sites was evaluated using a photochemically induced acute ischemic stroke model in mice, following previously reported protocols [47]. To track the nanoparticles *in vivo*, they were labeled with ICG and injected intravenously into the mice. The injected mice were imaged with an *in vivo* fluorescence imaging system. The results showed that PLPA@PFP could accumulate at the infarct site and reach a peak within 0.5 h (Figs. 4A, 4B and S5). By contrast, the rt-PA- or LPA@PFP-injected groups showed lower accumulation, indicating that PLPA@PFP had a good targeted effect *in vivo* and could rapidly target the infarct site. After 24 h, PLPA@PFP remained detectable at the infarct site and showed the most intense signal among the groups, demonstrating its sustained accumulation effect (Figs. 4A and 4C). To further substantiate the targeted accumulation of PLPA@PFP, brain tissues from the stroke mouse model were dissected and processed for immunofluorescence staining. As shown in Fig. 4D, elevated intravascular fibrinogen/fibrin was expressed in the ischemic regions, as compared with healthy mice, indicating the successful establishment of an acute ischemic stroke model. In the PLPA@PFP-injected group, the ischemic region showed a higher ICG fluorescence signal than the other groups, which overlapped with that of fibrinogen/fibrin (Fig. 4D, S6 and S7). These results further demonstrated the PLPA@PFP had a high specific affinity with thrombi *in vivo*. In addition, the half-life of PLPA@PFP in the blood was detected via fluorescence imaging. As shown in Figs. 4E and 4F, PLPA@PFP exhibited a prolonged half-life (t_1/2_ = 1.647 ± 0.86 h) compared with free rt-PA (t_1/2_ = 0.23 ± 0.14 h), which is beneficial for maintaining a therapeutic window and reducing the frequency of repeated injections.

### 3.4 Efficacy of femoral vein thrombolysis in a mouse model

**Fig. 5:**
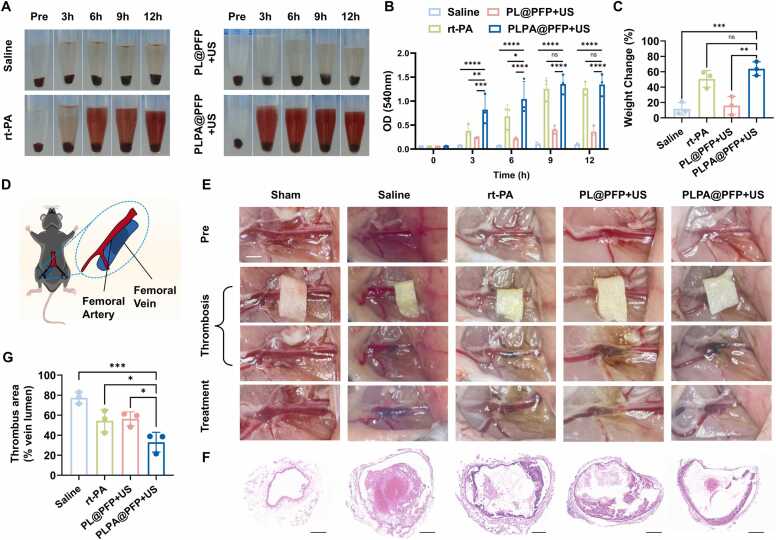
Assessment of dissolution efficiency in ex vivo blood clots and femoral vein thrombus in a mouse model. (A) Representative images of the thrombolysis process at 0, 3, 6, 9, and 12 h after treatment with saline, rt-PA, PL@PFP+US, and PLPA@PFP+US. (B) Absorbance values (OD 540 nm) of the supernatants at different time points after treatments. *n* = 3, mean ± SD; *p < 0.05, * *p < 0.01, * **p < 0.001, * ** *p < 0.0001. (C) Quantification of the dissolution efficiency by measuring the mass loss percentage of the blood clot at 12 h after treatment. *n* = 3, mean ± SD; * *p < 0.01, * **p < 0.001. (D) Establishing the FeCl_3_-induced femoral vein thrombus model. (E) Representative images of thrombolysis evaluation after treatment with saline, rt-PA, PL@PFP+US, and PLPA@PFP+US. Scale bar = 1 mm. (F) Histological analysis of the femoral vein after treatment with saline, rt-PA, PL@PFP+US, and PLPA@PFP+US for 12 h. Scale bar = 100 µm. (G) Quantification of thrombus area (% vein lumen) in the femoral vein in different treatment groups. *n* = 3, mean ± SD; *p < 0.05, * **p < 0.001.

Thrombolysis efficacy was evaluated using *ex vivo* blood clots, which were prepared from fresh mouse blood and thrombin (Fig. 5A). Four groups were established: saline, rt-PA, PL@PFP+US, and PLPA@PFP+US. The saline group represents untreated thrombosis, while the rt-PA group represents pharmacological thrombolysis. The PL@PFP+US group reflects sonothrombolysis triggered by ultrasound without rt-PA. The PLPA@PFP+US group combines pharmacological thrombolysis and sonothrombolysis. Focused US was applied for 10 min in the first 3 h. The dissolution efficiency was assessed by measuring hemoglobin levels (red color) in the supernatants every 3 h. The mass loss of the clots after 12 h was quantified to confirm thrombolytic effects. As shown in Figs. 5B and 5C, although there was no significant difference between the rt-PA and PLPA@PFP+US groups at the end of 12 h, PLPA@PFP+US treatment exhibited a notably higher dissolution efficiency within the initial 3 h, approximately three-fold higher than that with rt-PA treatment. This suggests that PLPA@PFP+US has rapid and synergistic dissolution efficiency of *ex vivo* blood clots, mainly due to sonothrombolysis and enhanced penetration of rt-PA facilitated by US.

We further conducted *in vivo* assessments of thrombolytic activity using a mouse model with femoral vein thrombus. The femoral vein was selected due to its larger caliber compared with the femoral artery, which made it easier to observe. Male C57BL/6 J mice were pretreated with 15 % FeCl_3_ to induce clot formation, followed by administration of PLPA@PFP through the tail vein and subsequent 10-min focused US treatment (Figs. 5D and 5E). Control groups included mice treated with saline, rt-PA, and PL@PFP+US. Thrombus images were recorded 12 h after initiating the treatments. As depicted in Fig. 5E, the femoral vein appeared dark at the sites where FeCl_3_-impregnated filter paper was applied, confirming successful thrombus formation. After 12 h, the PLPA@PFP+US group showed a superior thrombus dissolution effect compared with the other groups. Histological results (Fig. 5F and S8) of the femoral vein were consistent with the results shown in Fig. 5E. The thrombus area, expressed as a percentage of the vein lumen, was estimated by measuring the cross-sectional areas of clots and veins in the histological images. The estimated thrombus areas were found to be 77.36 % for the saline group, 54.36 % for the rt-PA group, 56.32 % for the PL@PFP+US group, and 32.98 % for the PLPA@PFP+US group (Fig. 5G). To verify the role of US in the thrombolysis process, ICG-labeled PLPA@PFP was injected after femoral thrombi formation, and fluorescence was detected using IVIS (Figure S9). The results showed that after US treatment, PLPA@PFP tended to accumulate inside the thrombus. In contrast, in the groups without US treatment, PLPA@PFP was more likely to accumulate around the periphery of the thrombus. This suggested that the US facilitates penetration of PLPA@PFP into the thrombus, potentially enhancing its thrombolytic effectiveness. These findings demonstrated that PLPA@PFP+US offers a more effective therapeutic strategy for restoring blood flow in occluded vessels.

### 3.5 In vivo thrombolysis effect of PLPA@PFP evaluated using high-resolution PA imaging

**Fig. 6:**
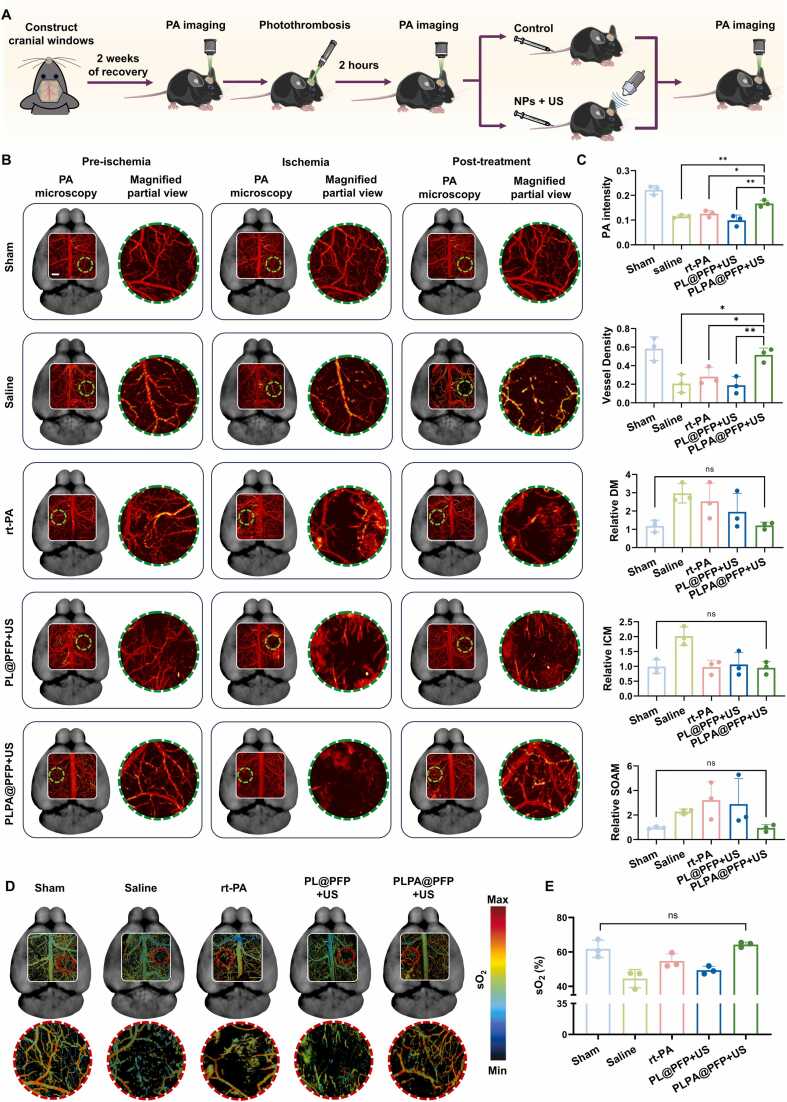
PA imaging evaluation of the therapeutic effect of PLPA@PFP in photothrombotic models. (A) Schematic illustration of the PAM imaging and treatment protocol. (B) PA images at pre-ischemia, ischemia (2 h), and post-treatment (day 3) stages of photothrombotic stroke. Scale bar= 500 µm. (C) Data analysis of vascular parameters response to the photothrombosis and treatment, including PA intensity, vessel density, vascular tortuosity, DM, ICM, and SOAM. *n* = 3, mean ± SD; *p < 0.05, * *p < 0.01. (D) Parametric map of estimated sO_2_ of different groups after 3 days of treatment. Scale bar= 500 µm. (E) Quantification of photoacoustic blood oxygen in (D). *n* = 3, mean ± SD, ns means no significance.

We prepared transparent cranial windows in healthy male C57BL/6 J mice (8–10 weeks old, 25–30 g), according to previous reports to monitor the cerebrovascular structure and metabolic activity[57]. In this work, cranial windows were constructed to prevent attenuation and distortion of the pulsed laser caused by the skull. In brief, a 5 mm × 6 mm rectangular bone flap was carefully removed and replaced with a transparent film (Figure S10). Following a 2-week recovery period, the operative incision and related inflammatory response to the cranial window installation had completely subsided. To evaluate the efficacy of PLPA@PFP treatment in a stroke model induced by photothrombosis, we further conducted PA imaging. This model was established by injecting rose bengal via the tail vein, followed by laser irradiation of the brain to induce thrombus formation[47]. Guided by PA imaging, a 1.5 mm circular area with an adequate number of microvessels was irradiated with a 532 nm laser (80 mW) for 5 min. After 2 h, the vascular PA signal in the irradiated area weakened or even diminished, indicating the successful induction of stroke. Then, the mice were treated with either saline or PLPA@PFP+US, and their recovery was evaluated after 3 d (Figs. 6A and 6B). A representative area within the green-dashed circle in Figure S11A (Supporting Information) was selected to assess the resolution and signal-to-background ratio (SBR) of PA imaging. The PA intensity profile of a microvessel in this area was recorded and analyzed using a Gaussian fit. As illustrated in Figure S11B (Supporting Information), the resolution, represented by the Gaussian-fitted full width at half maximum (FWHM), was 11.1 µm. The calculated SBR was 13.8 dB, indicating high resolution and good SBR for PA imaging. Several parameters including PA intensity, vessel density, and vascular tortuosity (relative to pre-ischemia) were quantified. As shown in Figs. 6B and 6C, compared with the control group, the PLPA@PFP+US group exhibited higher PA intensity and vessel density, indicating better blood flow restoration after treatment. Vascular tortuosity characterized by the distance metric (DM), inflection count metric (ICM), and sum of angles metric (SOAM) was further analyzed. These relative values (post-treatment/pre-ischemia) closer to 1 indicated that the blood vessels retained their original morphology. As shown in Fig. 6C, the tortuosity values of the sham group were all close to 1, while saline-treated group exhibited significant deviation, demonstrating that the vessels were occluded and showed a disordered structure after stroke. After treatment with PLPA@PFP+US, the values approached to 1, indicating effective vessel reopening. The rt-PA group and the PL@PFP+US group also showed varying degrees of deviation. As expected, after treatment of PLPA@PFP+US, the sO_2_ was significantly increased, as detected on PA imaging (Figs. 6D and 6E). This result was mainly due to the restoration of vessel patency, enhancing the local oxygen supply and potentially mitigating anoxia-induced brain injury. Therefore, PA imaging provides direct insights into the hemodynamics of brain lesions, allowing for the observation of significant differences in sO_2_ between infarcted and normal brain areas. This ability to capture functional parameters makes it a valuable tool for the diagnosis and monitoring of ischemic stroke. Although the PA imaging is non-invasive, the invasive cranial windows are required to assist PA imaging. Therefore, future developments in PA imaging technology that enable efficient transcranial imaging will be crucial for non-invasive clinical monitoring and assessment of cerebrovascular structures and functions.

### 3.6 Thrombolysis effect of PLPA@PFP in photothrombotic stroke model

**Fig. 7:**
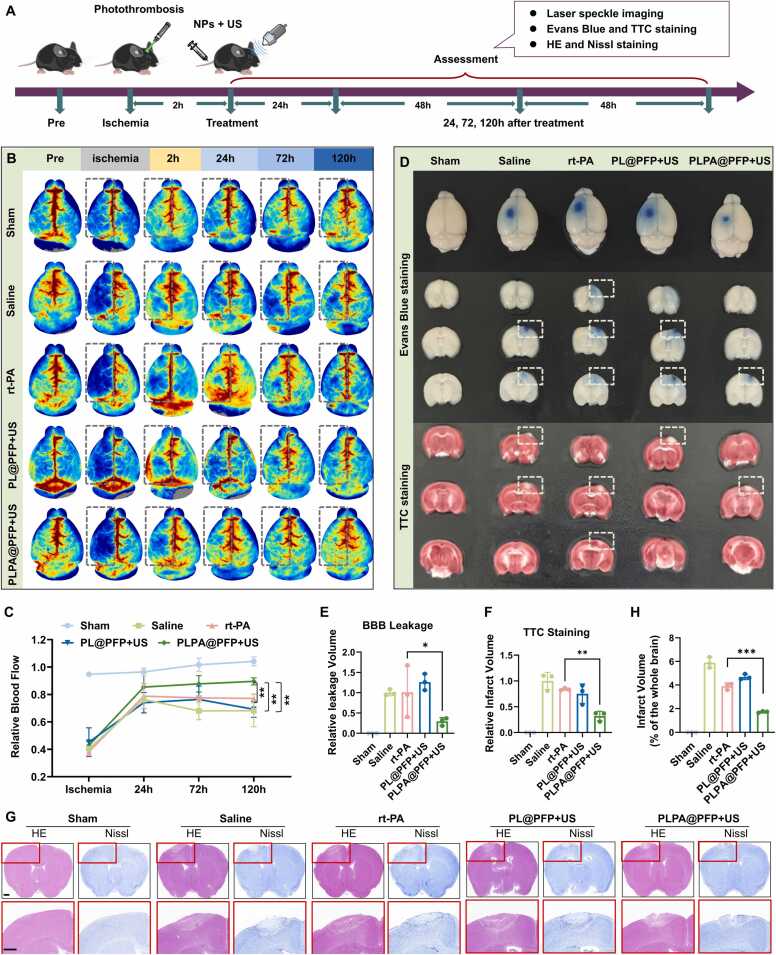
In vivo thrombolysis evaluation in ischemic stroke models. (A) Experimental scheme of thrombosis model construction, antithrombotic treatment, and thrombolytic effect evaluation. (B) Representative laser speckle images taken from representative mice in sham, saline-, rt-PA-, PL@PFP+US-, and PLPA@PFP+US-treated groups. (C) Data analysis of the relative blood flow monitored by laser speckle in sham, saline-, rt-PA-, PL@PFP+US-, and PLPA@PFP+US-treated groups. *n* = 3, mean ± SD; * *p < 0.01. (D) Representative Evans Blue and TTC staining images of stroke mouse brain 5-day post-administration for measuring permeability of the BBB and infarct volume in sham, saline-, rt-PA-, PL@PFP+US-, and PLPA@PFP+US-treated groups. (E) Data analysis of BBB permeability in stroke mouse brain 5 days after treatment. *n* = 3, mean ± SD; *p < 0.05. (F) Data analysis of infarct volume in stroke mouse brain 5 days after treatment. n = 3, mean ± SD; * *p < 0.01. (G) Representative HE and Nissl staining images of cerebral infarct area in sham, saline-, rt-PA-, PL@PFP+US-, and PLPA@PFP+US-treated groups. Scale bar= 500 µm. (H) Data analysis of infarct volume in sham, saline-, rt-PA-, PL@PFP+US-, and PLPA@PFP+US-treated groups. *n* = 3, mean ± SD; * **p < 0.001.

The thrombolysis effect of PLPA@PFP *in vivo* was fully assessed using conventional methods. As shown in the treatment schedule (Fig. 7A), at 2 h post-ischemia, the mice were treated with saline, rt-PA, PL@PFP+US, or PLPA@PFP+US. Focal cerebral blood flow was monitored before and after treatment using a laser speckle imaging system. As shown in Figs. 7B and 7C, blood flow in the occluded areas was significantly reduced after laser excitation. After treatment, the PLPA@PFP+US group showed a marked restoration of cerebral blood flow at 24 h compared with the other groups, and the ischemic area continued to decrease over the following days. By contrast, the saline-treated group exhibited a continued decline in focal blood flow over 72 h, likely due to edema induced by cerebral infarction. An enlarged area of edema typically occurs within 72 h after a stroke, exerting compression on adjacent tissues, thereby adversely affecting local blood circulation[58]. Rapid thrombolysis with PLPA@PFP+US facilitated the prevention of secondary edema in stroke.

At 5 days post-treatment, the permeability of the blood–brain barrier (BBB) was assessed using Evans blue staining, and the infarct volume was detected by TTC staining according to previous reports[59], [60]. As indicated by the color intensity, PLPA@PFP+US treatment significantly reduced the BBB permeability and infarct volume, compared with the saline, rt-PA, and PL@PFP+US groups (Figs. 7D, 7E and 7F). Hematoxylin & eosin (HE) and Nissl staining also validated that the cerebral infarct area in the PLPA@PFP+US group was much smaller than that in the other groups (Figs. 7G and 7H), which was consistent with the results of laser speckle imaging and TTC staining. These findings demonstrated that PLPA@PFP+US could effectively restore focal cerebral blood flow, reduce infarct volume, and preserve BBB integrity, primarily due to its rapid and synergistic thrombolytic effect. The efficient treatment effect was mainly due to: 1) PLPA@PFP selectively targeting activated platelets within the thrombus, enhancing the local concentration of rt-PA at the clot site, thereby improving thrombolysis efficacy while minimizing systemic exposure and off-target effects; and 2) Mechanical forces generated by ultrasound and PLPA@PFP disrupting the clot structure, making it more permeable to rt-PA and accelerating clot breakdown.

### 3.7 Biosafety evaluation

The biosafety of PLPA@PFP was assessed *in vitro* and *in vivo*. As shown in Figure S12, the cell viability of HUVECs had no significant decrease when treated with PLPA@PFP at the concentration up to 400 μg/mL for 24 hours. Red blood cells treated with PLPA@PFP at the concentration up to 200 μg/mL for 4 hours showed negligible hemolysis (hemolytic ratio <2 %) (Figure S13). These results demonstrated that PLPA@PFP has less cytotoxicity and good hemocompatibility. Additionally, we monitored the biodistribution of PLPA@PFP in major organs including heart, liver, spleen, lung, kidney, pancreas and intestine. Fluorescence imaging was performed at 1, 24, and 48 h post-injection (Figure S14). At 1 h, fluorescence signals were observed in the brain (infarct area), liver, kidney, intestine and lung, indicating initial distribution. The transient presence of NPs in the lungs shortly after administration is consistent with normal circulation patterns following intravenous injection, because even with standard rt-PA injections, residual signals in the lungs can be observed. By 24 h, fluorescence in these organs decreased significantly, and by 48 h, it was nearly undetectable. This suggests that PLPA@PFP could be cleared from the body within 48 h. The hematological analyses of healthy mice and stroke models treated with saline, rt-PA, and PLPA@PFP were conducted as expected. The results showed no statistical differences between the healthy and PLPA@PFP treated group (Figure S15). Histopathological evaluation of major organs (heart, liver, spleen, lungs, and kidneys) stained with hematoxylin and eosin showed no pathological changes in either the treatment or control groups (Figure S16).

These findings collectively affirm the safety of PLPA@PFP *in vitro* and *in vivo*, highlighting its potential translation value for stroke therapy. The platelet membrane coating enables precise targeting at thrombus sites, improving therapeutic accuracy while minimizing off-target effects. The combination of this system with sonothrombolysis has shown strong therapeutic efficacy in our study, which could potentially lead to improved clinical outcomes in stroke patients. However, several challenges remain for clinical translation. Firstly, the manufacturing process is complex and demands stringent purity control, as producing nanoparticles with consistent quality at scale is difficult. Impurities could potentially trigger immune responses, increase toxicity, and reduce targeting efficiency. Secondly, the clinical application of PLPA@PFP+US therapy requires specialized ultrasound equipment and precise control of ultrasound parameters to ensure targeted delivery without harming surrounding tissues. Addressing these challenges will be critical in future studies.

## 4 Conclusions

In summary, we developed a new approach for efficient and targeted thrombolysis in cases of ischemic stroke, using high-resolution PA image-guided bioinspired nanoparticles. In this novel approach, both PFP and rt-PA are delivered by a platelet-biomimetic membrane system (PLPA@PFP). PLPA@PFP was found to rapidly target and accumulate at the thrombus site. By integrating rt-PA-mediated drug thrombolysis with US-induced PFP cavitation, PLPA@PFP had a potent and safe thrombolytic effect in ischemic stroke models. The efficacy of these treatments was confirmed through high-resolution PA imaging and laser speckle imaging, which provides detailed insight into the changes in vascular structure, blood-oxygen and bloodstream within the infarct area. In addition, PLPA@PFP exhibited excellent compatibility and biosafety, both *in vitro* and *in vivo*. This study introduces a groundbreaking therapeutic strategy that not only ensures rapid clot dissolution but also utilizes PA imaging to monitor microvessel structure and sO_2_, without the need for probe labeling. Given its potential, this approach holds promise for future preclinical and clinical applications in stroke management.

## CRediT authorship contribution statement

**Liu Chengbo:** Writing – review & editing, Supervision, Funding acquisition, Conceptualization. **Yu Mengchen:** Software, Methodology, Data curation. **Xue Zhiwei:** Writing – original draft, Methodology, Investigation. **Chen Jingqin:** Writing – review & editing, Supervision, Funding acquisition, Conceptualization. **Han Mengtao:** Writing – original draft, Methodology, Investigation, Data curation, Conceptualization. **Wang Donghai:** Writing – review & editing, Supervision, Funding acquisition, Conceptualization. **Wu Zhifeng:** Visualization, Methodology, Formal analysis, Data curation. **Xu Zhiqiang:** Software, Methodology. **Ren Yaguang:** Software, Methodology, Investigation. **You Nanlin:** Validation, Software, Methodology, Data curation. **Sheng Zonghai:** Writing – review & editing, Supervision. **He Yiming:** Validation, Methodology, Investigation.

## Declaration of Competing Interest

The authors declare that they have no known competing financial interests or personal relationships that could have appeared to influence the work reported in this paper.

## Data Availability

Data will be made available on request.
