# Supplementary material for: Rapid synergistic thrombolysis of ischemic stroke guided by high-resolution and high-speed photoacoustic cerebrovascular imaging
Source: Photoacoustics. 2025 Apr 4;43:100722. doi: 10.1016/j.pacs.2025.100722 (PMC12018002; doi:10.1016/j.pacs.2025.100722)
Supplement: Supplementary file 1 — Supplementary material [file mmc1.docx]

**Supplementary Data**

**Rapid Synergistic Thrombolysis of Ischemic Stroke Guided by High-Resolution** **and High-Speed Photoacoustic Cerebrovascular Imaging**

Mengtao Han ^a, b, d, 1^, Zhiwei Xue ^a, d, 1^, Mengchen Yu ^a, d^, Nanlin You ^a, d^, Yaguang Ren ^b, c^, Zhiqiang Xu ^b, c^, Zhifeng Wu ^b, c^, Yiming He ^a, d^, Zonghai Sheng ^c, f^, Chengbo Liu ^b, c, *^, Donghai Wang ^a, d, e, *^ and Jingqin Chen ^b, c, *^

^a^ Department of Neurosurgery, Qilu Hospital of Shandong University, Cheeloo College of Medicine and Institute of Brain and Brain-Inspired Science, Shandong University, Jinan 250012, China

^b^ Research Center for Biomedical Optics and Molecular Imaging, Shenzhen Institute of Advanced Technology, Chinese Academy of Sciences, Shenzhen 518055, China

^c^ State Key Laboratory of Biomedical Imaging Science and System, Shenzhen 518055, China

^d^ Shandong Key Laboratory of Brain Health and Function Remodeling, Jinan 250012, China

^e^ Department of Neurosurgery, Qilu Hospital of Shandong University Dezhou Hospital, Dezhou 253000, China

^f^ Research Center for Advanced Detection Materials and Medical Imaging Devices, Institute of Biomedical and Health Engineering, Shenzhen Institute of Advanced Technology, Chinese Academy of Sciences, Shenzhen 518055, China

* Corresponding authors.

*E-mail addresses:* cb.liu@siat.ac.cn (C. Liu); drwangdonghai@sdu.edu.cn (D. Wang); jq.chen@siat.ac.cn (J. Chen)

^1^ These authors contributed equally to this work.
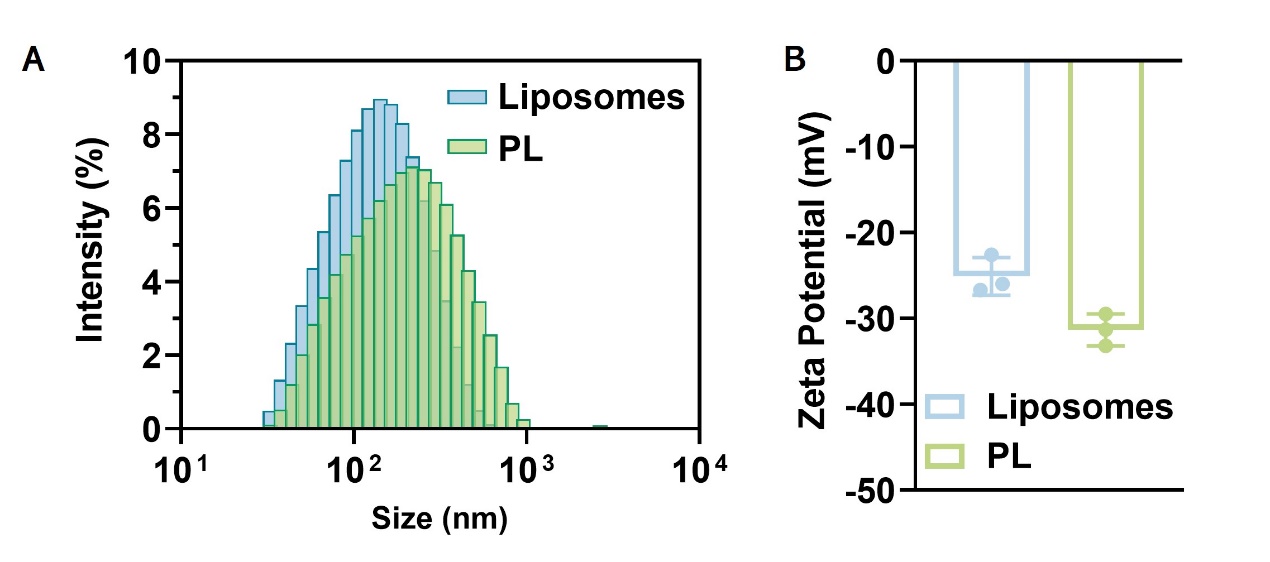


**Figure S1.** (A) Diameter and (B) zeta potential of Liposomes and PL.


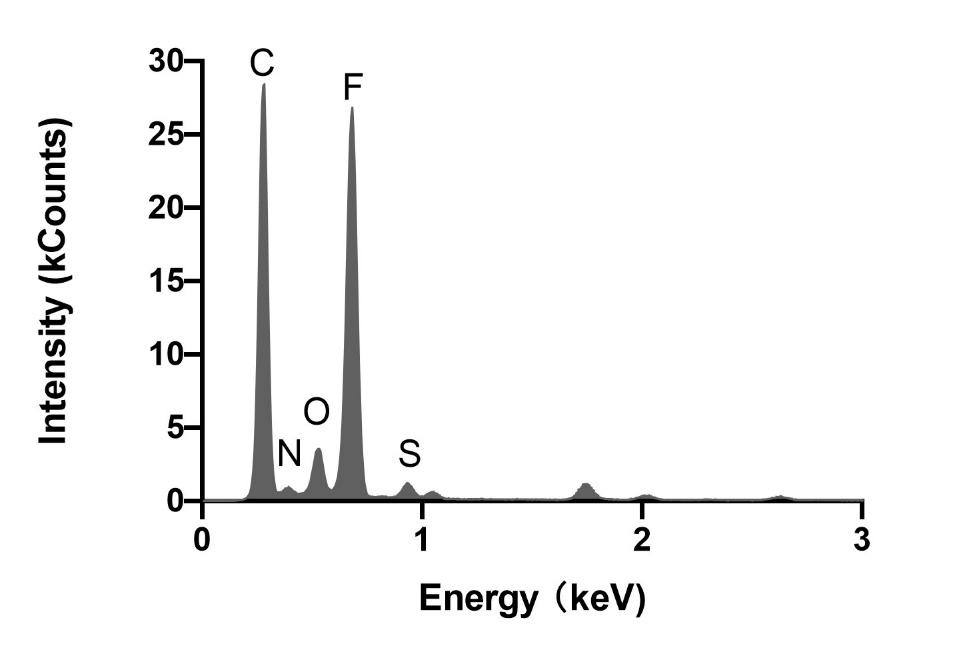


**Figure S2.** Analysis of element mappings of C, N, O, F, S signals of PLPA@PFP.

| Concentration of PLPA | Concentration of rt-PA | Drug-loading efficiency |
| --- | --- | --- |
| 5mg/mL | 2.324mg/mL | 46.48% |

**Table S1.** Loading efficiency of rt-PA.


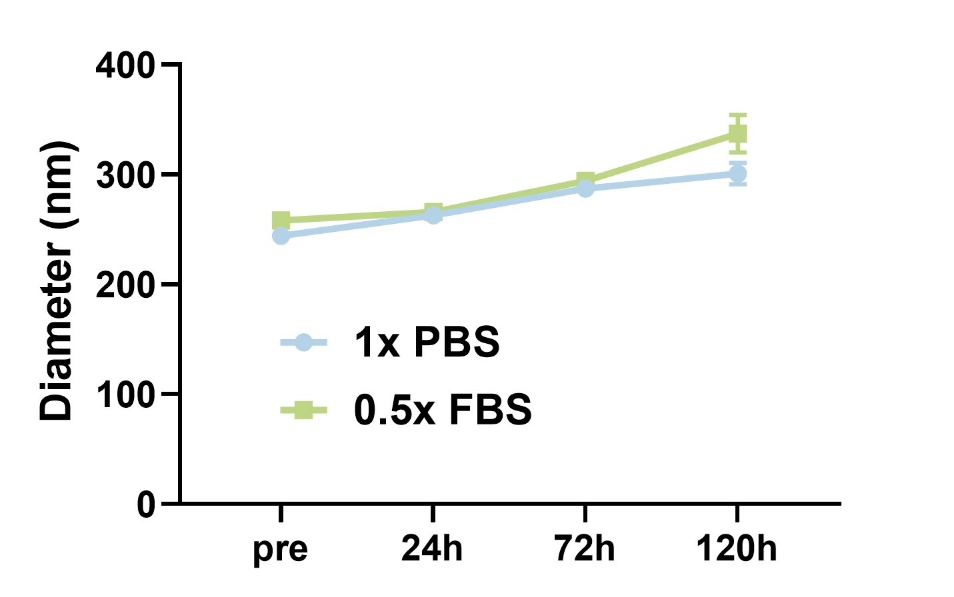


**Figure S3.** Changes of nanoparticle size of PLPA in 1× PBS or 0.5× FBS over 120h.


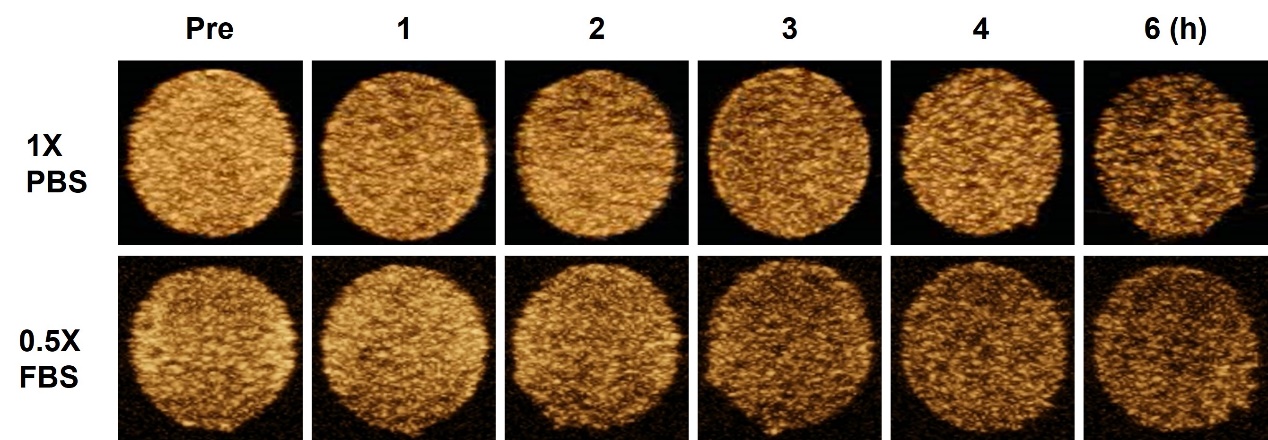


**Figure S4.** Changes of ultrasound signal of PLPA@PFP in 1× PBS or 0.5× FBS over 6h.
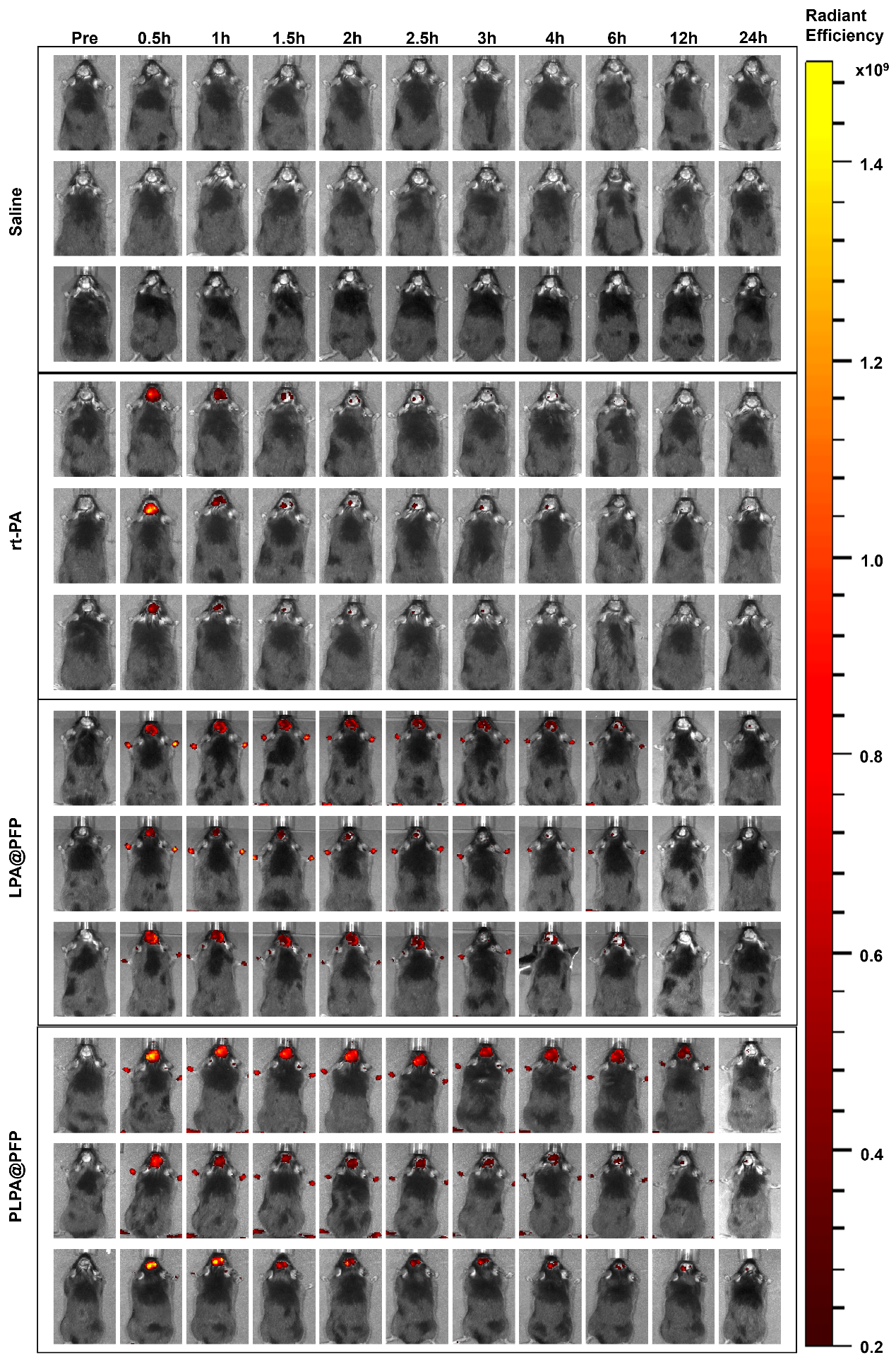


**Figure S5.** Representative fluorescence images of brain of all animals after injection of saline, ICG-labeled rt-PA, LPA@PFP and PLPA@PFP within 24h.


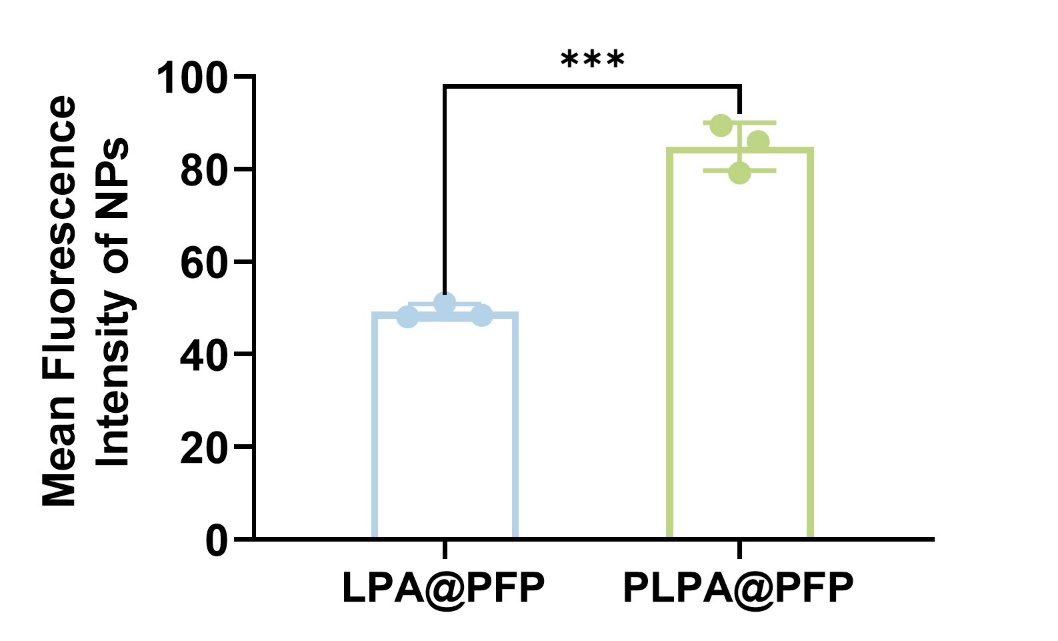


**Figure S6.** Mean fluorescence intensity of LPA@PFP and PLPA@PFP in Figure 4D. *n* = 3, mean ± SD; ****p* < 0.001.


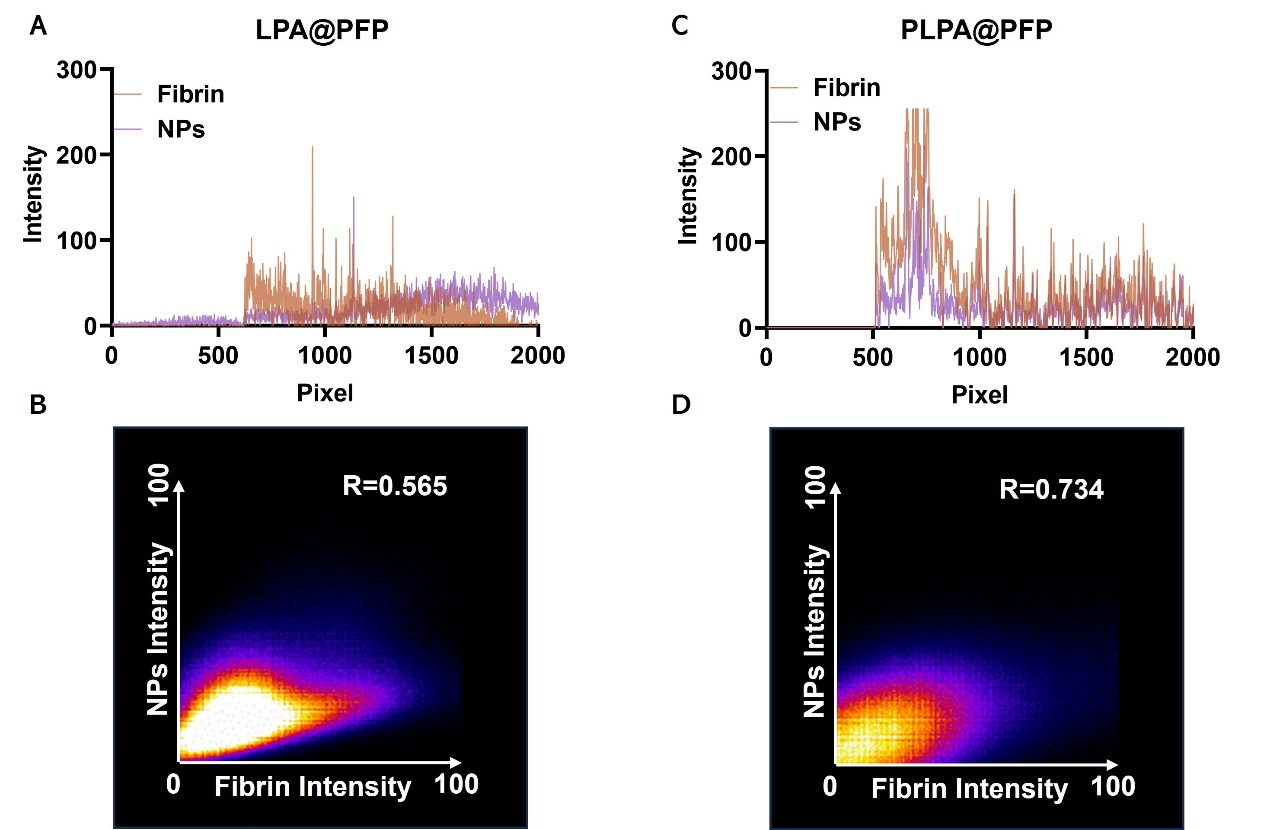


**Figure S7.** (A) and (C), the fluorescence intensity plot profile of NPs with fibrin. (B) and (D), co-localizations of PLPA@PFP NPs with fibrin in confocal images.


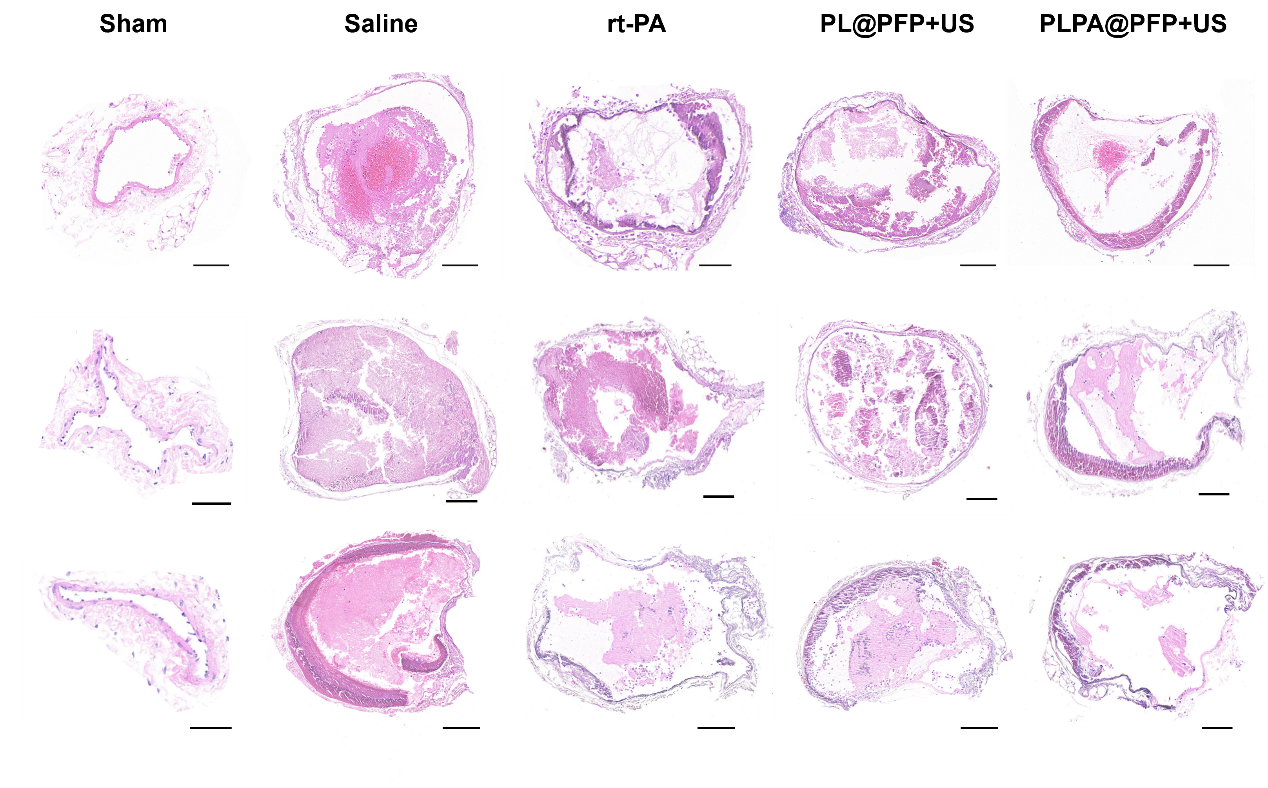


**Figure S8.** Histological analysis of the femoral vein after treatment with saline, rt-PA, PL@PFP+US, and PLPA@PFP+US for 12 h. Scale bar=100µm.


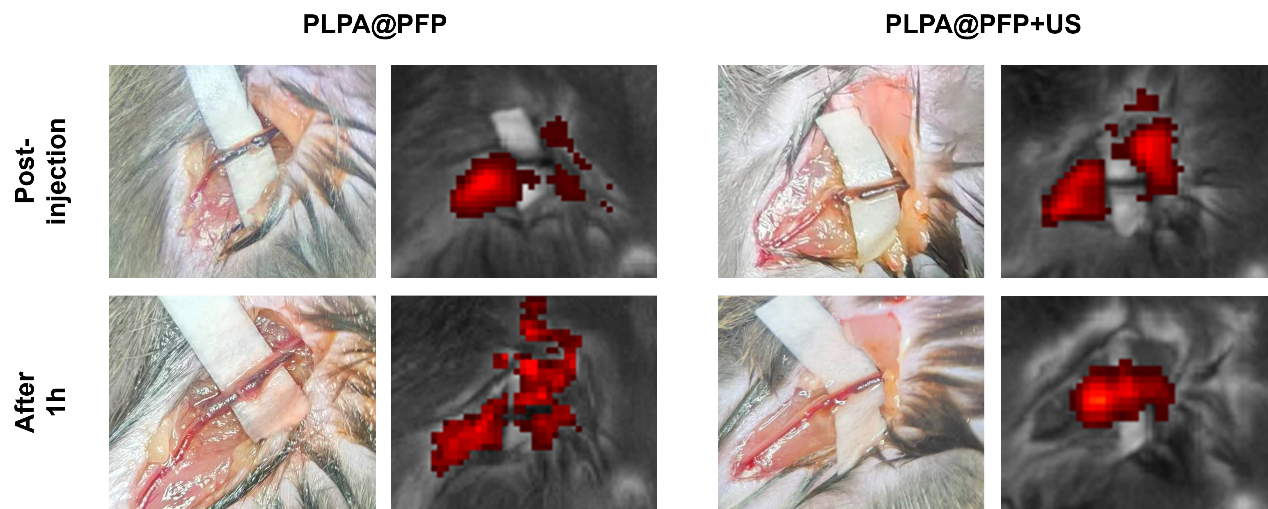


**Figure S9.** US enhances the permeability of PLPA@PFP in femoral thrombi. Representative images of PLPA@PFP (labeled with ICG) accumulation at the thrombus site.


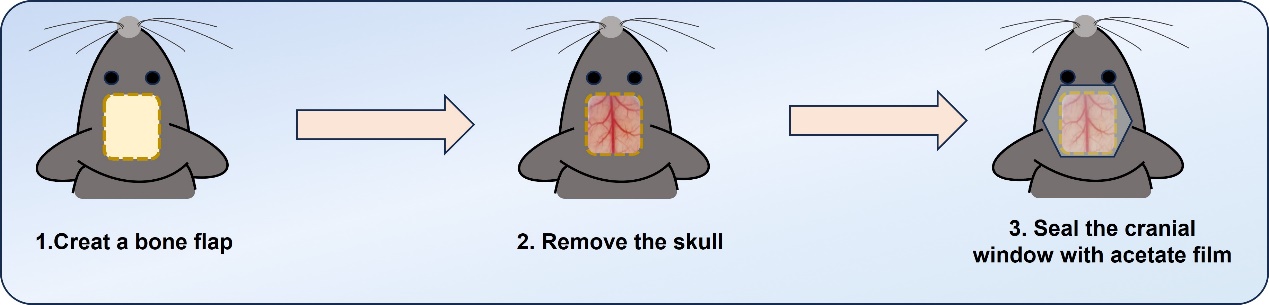


**Figure S10.** Schematic illustration of the procedure of construction of cranial windows.


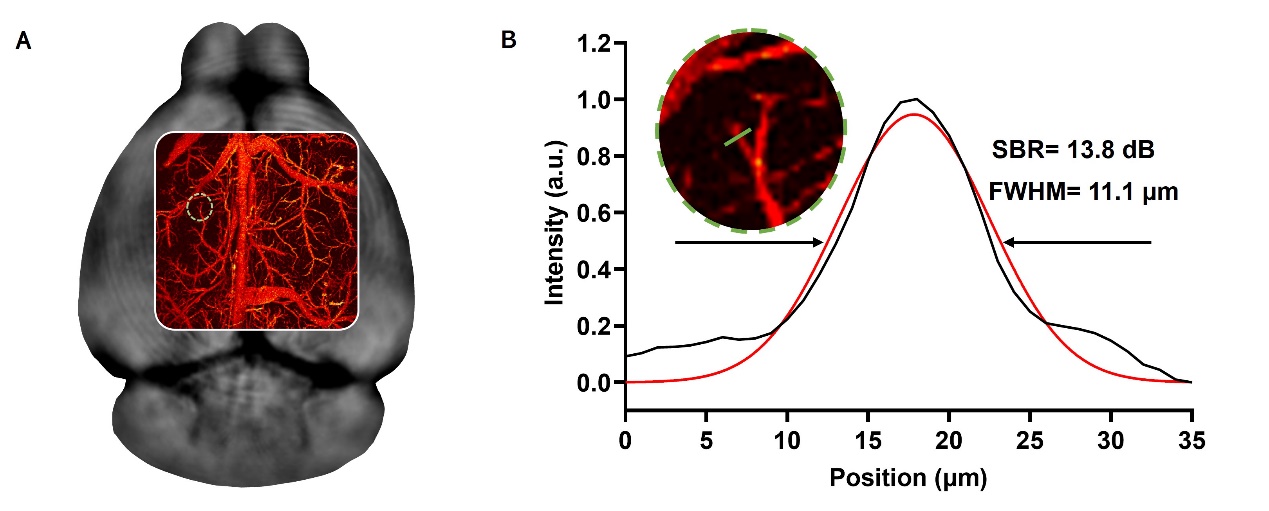
**Figure S11.** (A) PA image of pre-treatment in saline group. (B) PA intensity profile (black curve) along the green line in the zoomed area (insets in [B]), which was selected from (A) (green dashed circle). Gaussian fits to the profiles are described with red curves, and FWHM of the selected vessel along the green line is presented.


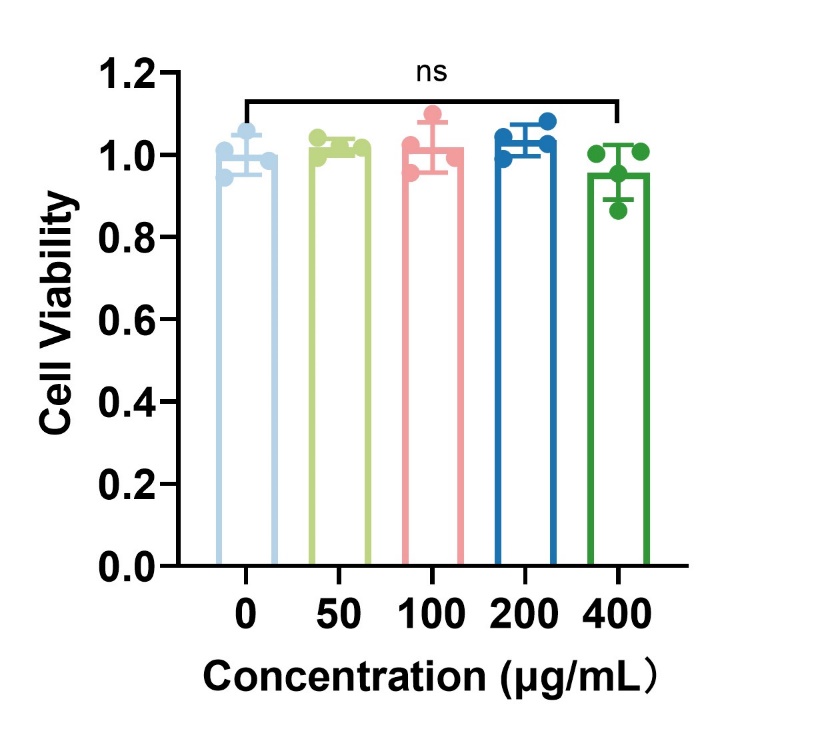


**Figure S12.** Cytotoxicity of PLPA@PFP to HUVECs. n = 3, mean ± SD; ns means no significant difference.


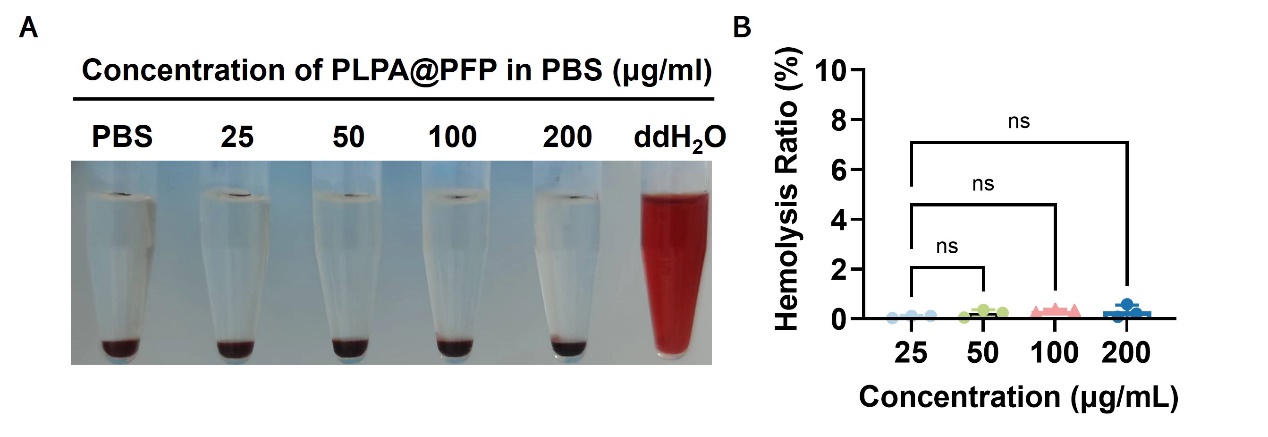


**Figure S13.** (A) Hemolysis test and (B) data analysis of PLPA@PFP nanoparticles. *n* = 3, mean ± SD; ns means no significant difference.


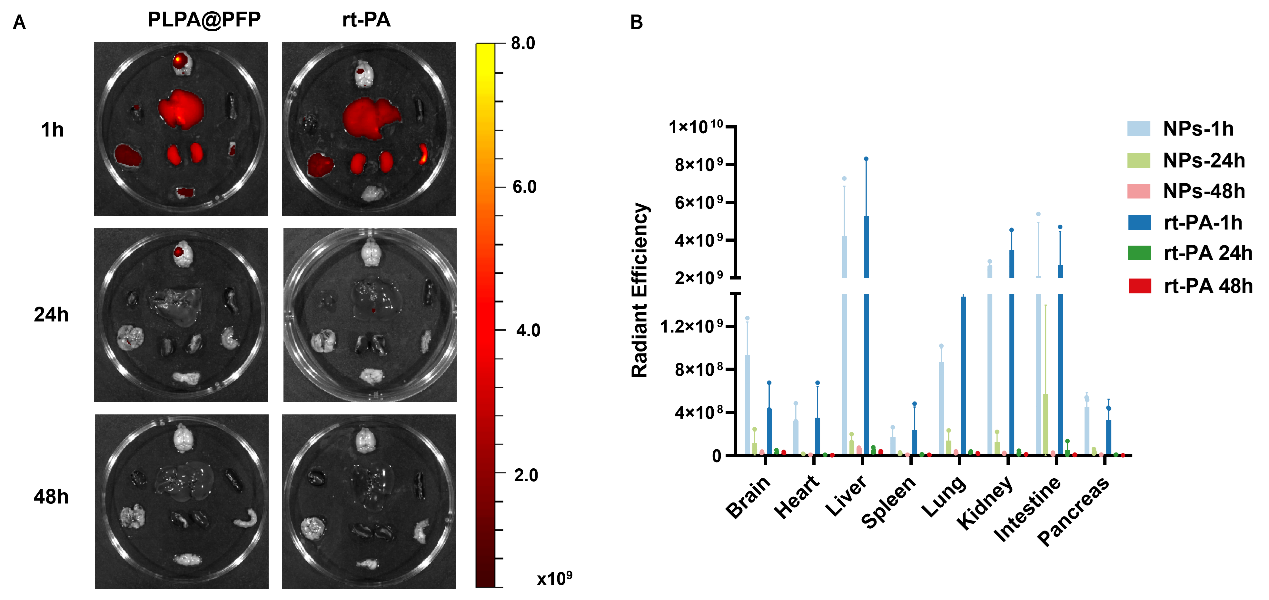


**Figure S14.** (A) Biodistribution and (B) data analysis of ICG labeled PLPA@PFP and rt-PA in major organs (including heart, brain, spleen, lung, liver, kidney, intestine and pancreas) after injection into stroke mice within 48 h (*n* = 3, mean ± SD).


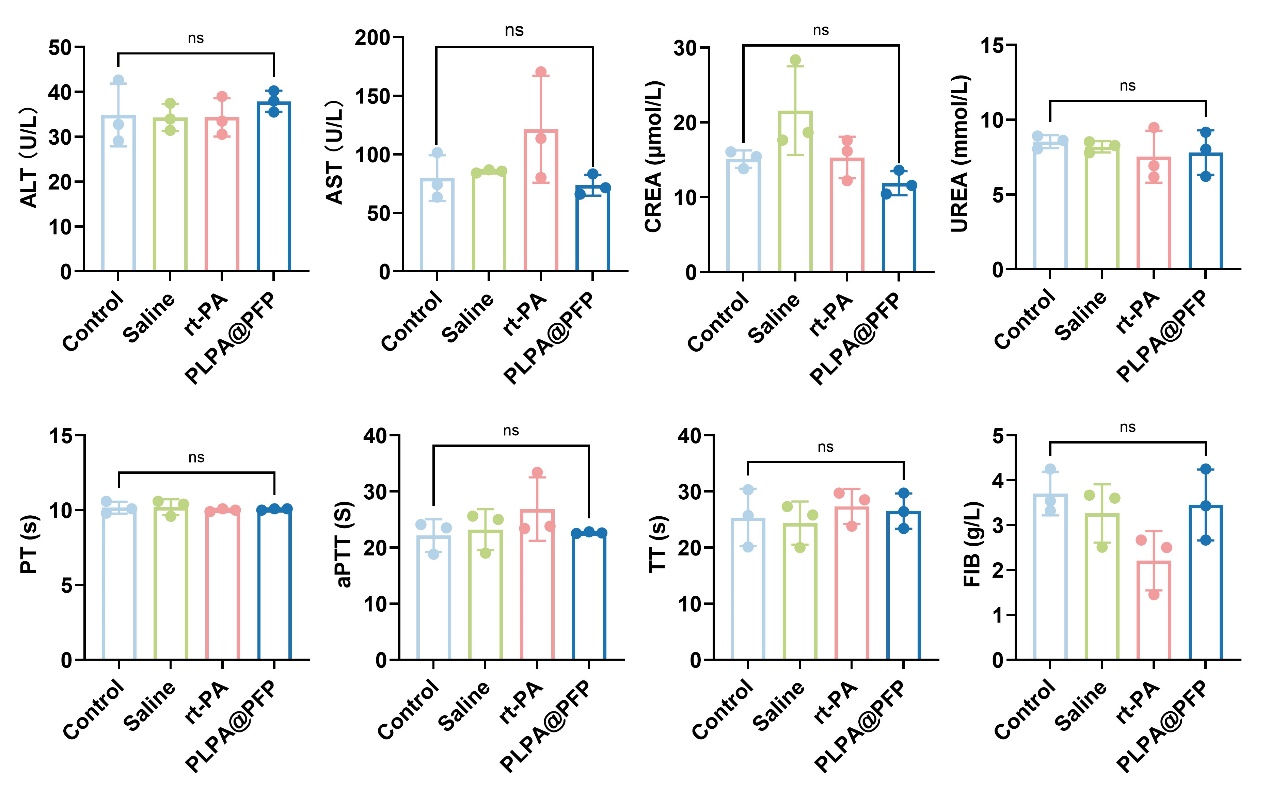


**Figure S15.** Typical indicators of liver and kidney function, including ALT, AST, CREA and UREA and parameters of coagulation (aPTT, FIB, PT, and TT) post administration with different treatment. *n* = 3, mean ± SD; ns means no significant difference.


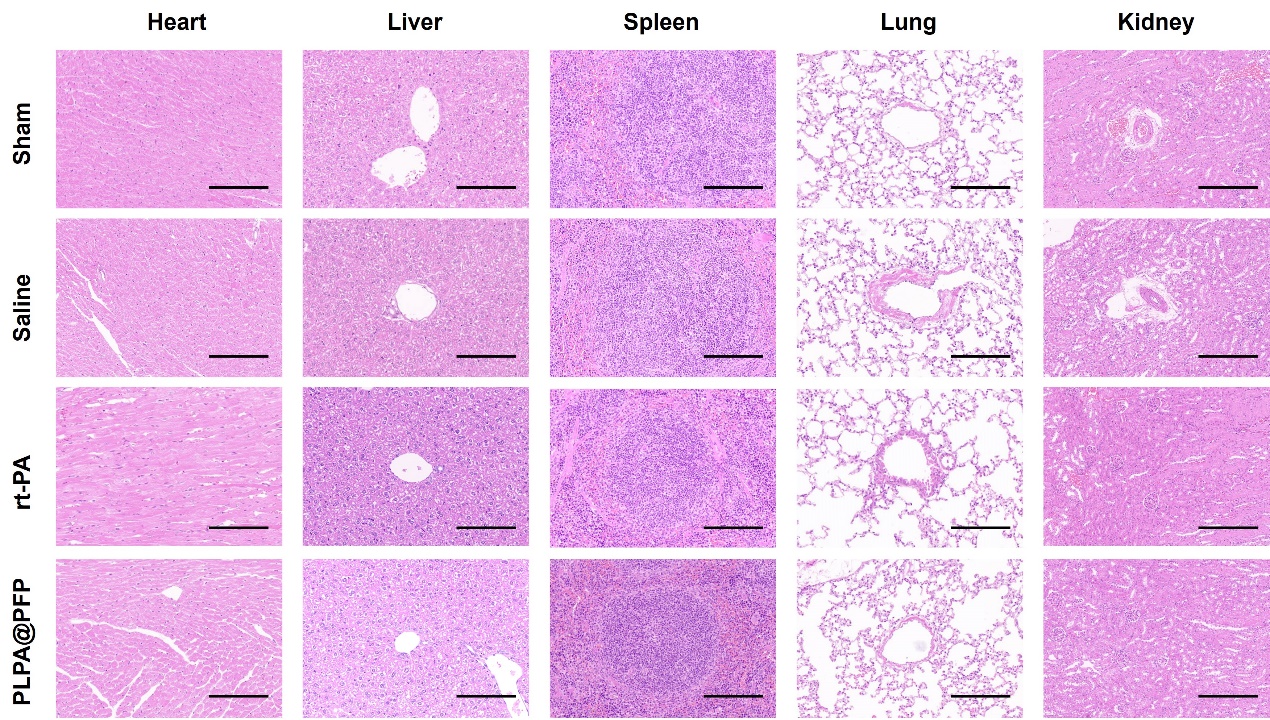


**Figure S16.** H&E staining images of major organs in sham group and stroke model mice treated with saline, rt-PA, PLPA@PFP. Scale bar=100µm.
